# Genome assembly and resequencing analyses provide new insights into the evolution, domestication and ornamental traits of crape myrtle

**DOI:** 10.1093/hr/uhad146

**Published:** 2023-07-21

**Authors:** Yang Zhou, Tangchun Zheng, Ming Cai, Lu Feng, Xiufeng Chi, Ping Shen, Xin Wang, Zhiting Wan, Cunquan Yuan, Man Zhang, Yu Han, Jia Wang, Huitang Pan, Tangren Cheng, Qixiang Zhang

**Affiliations:** Beijing Key Laboratory of Ornamental Plants Germplasm Innovation & Molecular Breeding, National Engineering Research Center for Floriculture, Beijing Laboratory of Urban and Rural Ecological Environment, Engineering Research Center of Landscape Environment of Ministry of Education, Key Laboratory of Genetics and Breeding in Forest Trees and Ornamental Plants of Ministry of Education, School of Landscape Architecture, Beijing Forestry University, Beijing 100083, China; Beijing Key Laboratory of Ornamental Plants Germplasm Innovation & Molecular Breeding, National Engineering Research Center for Floriculture, Beijing Laboratory of Urban and Rural Ecological Environment, Engineering Research Center of Landscape Environment of Ministry of Education, Key Laboratory of Genetics and Breeding in Forest Trees and Ornamental Plants of Ministry of Education, School of Landscape Architecture, Beijing Forestry University, Beijing 100083, China; Beijing Key Laboratory of Ornamental Plants Germplasm Innovation & Molecular Breeding, National Engineering Research Center for Floriculture, Beijing Laboratory of Urban and Rural Ecological Environment, Engineering Research Center of Landscape Environment of Ministry of Education, Key Laboratory of Genetics and Breeding in Forest Trees and Ornamental Plants of Ministry of Education, School of Landscape Architecture, Beijing Forestry University, Beijing 100083, China; Beijing Key Laboratory of Ornamental Plants Germplasm Innovation & Molecular Breeding, National Engineering Research Center for Floriculture, Beijing Laboratory of Urban and Rural Ecological Environment, Engineering Research Center of Landscape Environment of Ministry of Education, Key Laboratory of Genetics and Breeding in Forest Trees and Ornamental Plants of Ministry of Education, School of Landscape Architecture, Beijing Forestry University, Beijing 100083, China; Beijing Key Laboratory of Ornamental Plants Germplasm Innovation & Molecular Breeding, National Engineering Research Center for Floriculture, Beijing Laboratory of Urban and Rural Ecological Environment, Engineering Research Center of Landscape Environment of Ministry of Education, Key Laboratory of Genetics and Breeding in Forest Trees and Ornamental Plants of Ministry of Education, School of Landscape Architecture, Beijing Forestry University, Beijing 100083, China; Beijing Key Laboratory of Ornamental Plants Germplasm Innovation & Molecular Breeding, National Engineering Research Center for Floriculture, Beijing Laboratory of Urban and Rural Ecological Environment, Engineering Research Center of Landscape Environment of Ministry of Education, Key Laboratory of Genetics and Breeding in Forest Trees and Ornamental Plants of Ministry of Education, School of Landscape Architecture, Beijing Forestry University, Beijing 100083, China; Beijing Key Laboratory of Ornamental Plants Germplasm Innovation & Molecular Breeding, National Engineering Research Center for Floriculture, Beijing Laboratory of Urban and Rural Ecological Environment, Engineering Research Center of Landscape Environment of Ministry of Education, Key Laboratory of Genetics and Breeding in Forest Trees and Ornamental Plants of Ministry of Education, School of Landscape Architecture, Beijing Forestry University, Beijing 100083, China; Beijing Key Laboratory of Ornamental Plants Germplasm Innovation & Molecular Breeding, National Engineering Research Center for Floriculture, Beijing Laboratory of Urban and Rural Ecological Environment, Engineering Research Center of Landscape Environment of Ministry of Education, Key Laboratory of Genetics and Breeding in Forest Trees and Ornamental Plants of Ministry of Education, School of Landscape Architecture, Beijing Forestry University, Beijing 100083, China; Beijing Key Laboratory of Ornamental Plants Germplasm Innovation & Molecular Breeding, National Engineering Research Center for Floriculture, Beijing Laboratory of Urban and Rural Ecological Environment, Engineering Research Center of Landscape Environment of Ministry of Education, Key Laboratory of Genetics and Breeding in Forest Trees and Ornamental Plants of Ministry of Education, School of Landscape Architecture, Beijing Forestry University, Beijing 100083, China; Beijing Key Laboratory of Ornamental Plants Germplasm Innovation & Molecular Breeding, National Engineering Research Center for Floriculture, Beijing Laboratory of Urban and Rural Ecological Environment, Engineering Research Center of Landscape Environment of Ministry of Education, Key Laboratory of Genetics and Breeding in Forest Trees and Ornamental Plants of Ministry of Education, School of Landscape Architecture, Beijing Forestry University, Beijing 100083, China; Beijing Key Laboratory of Ornamental Plants Germplasm Innovation & Molecular Breeding, National Engineering Research Center for Floriculture, Beijing Laboratory of Urban and Rural Ecological Environment, Engineering Research Center of Landscape Environment of Ministry of Education, Key Laboratory of Genetics and Breeding in Forest Trees and Ornamental Plants of Ministry of Education, School of Landscape Architecture, Beijing Forestry University, Beijing 100083, China; Beijing Key Laboratory of Ornamental Plants Germplasm Innovation & Molecular Breeding, National Engineering Research Center for Floriculture, Beijing Laboratory of Urban and Rural Ecological Environment, Engineering Research Center of Landscape Environment of Ministry of Education, Key Laboratory of Genetics and Breeding in Forest Trees and Ornamental Plants of Ministry of Education, School of Landscape Architecture, Beijing Forestry University, Beijing 100083, China; Beijing Key Laboratory of Ornamental Plants Germplasm Innovation & Molecular Breeding, National Engineering Research Center for Floriculture, Beijing Laboratory of Urban and Rural Ecological Environment, Engineering Research Center of Landscape Environment of Ministry of Education, Key Laboratory of Genetics and Breeding in Forest Trees and Ornamental Plants of Ministry of Education, School of Landscape Architecture, Beijing Forestry University, Beijing 100083, China; Beijing Key Laboratory of Ornamental Plants Germplasm Innovation & Molecular Breeding, National Engineering Research Center for Floriculture, Beijing Laboratory of Urban and Rural Ecological Environment, Engineering Research Center of Landscape Environment of Ministry of Education, Key Laboratory of Genetics and Breeding in Forest Trees and Ornamental Plants of Ministry of Education, School of Landscape Architecture, Beijing Forestry University, Beijing 100083, China; Beijing Key Laboratory of Ornamental Plants Germplasm Innovation & Molecular Breeding, National Engineering Research Center for Floriculture, Beijing Laboratory of Urban and Rural Ecological Environment, Engineering Research Center of Landscape Environment of Ministry of Education, Key Laboratory of Genetics and Breeding in Forest Trees and Ornamental Plants of Ministry of Education, School of Landscape Architecture, Beijing Forestry University, Beijing 100083, China

## Abstract

Crape myrtle (*Lagerstroemia indica*) is a globally used ornamental woody plant and is the representative species of *Lagerstroemia*. However, studies on the evolution and genomic breeding of *L. indica* have been hindered by the lack of a reference genome. Here we assembled the first high-quality genome of *L. indica* using PacBio combined with Hi-C scaffolding to anchor the 329.14-Mb genome assembly into 24 pseudochromosomes. We detected a previously undescribed independent whole-genome triplication event occurring 35.5 million years ago in *L. indica* following its divergence from *Punica granatum*. After resequencing 73 accessions of *Lagerstroemia*, the main parents of modern crape myrtle cultivars were found to be *L. indica* and *L. fauriei*. During the process of domestication, genetic diversity tended to decrease in many plants, but this was not observed in *L. indica*. We constructed a high-density genetic linkage map with an average map distance of 0.33 cM. Furthermore, we integrated the results of quantitative trait locus (QTL) using genetic mapping and bulk segregant analysis (BSA), revealing that the major-effect interval controlling internode length (IL) is located on chr1, which contains *CDL15*, *CRG98*, and *GID1b1* associated with the phytohormone pathways. Analysis of gene expression of the red, purple, and white flower-colour flavonoid pathways revealed that differential expression of multiple genes determined the flower colour of *L. indica*, with white flowers having the lowest gene expression. In addition, BSA of purple- and green-leaved individuals of populations of *L. indica* was performed, and the leaf colour loci were mapped to chr12 and chr17. Within these intervals, we identified *MYB35*, *NCED*, and *KAS1*. Our genome assembly provided a foundation for investigating the evolution, population structure, and differentiation of Myrtaceae species and accelerating the molecular breeding of *L. indica*.

## Introduction

Crape myrtle (*Lagerstroemia indica*), the representative species of the *Lagerstroemia* genus, is a deciduous shrub or small tree with a long flowering period in summer and is one of the most beloved, iconic trees in tropical and warm-temperate regions. According to the *Flora of China*, 55 species belong to *Lagerstroemia*, of which 15 species (eight endemic) are distributed in China [1]. Crape myrtle originated in Southeast Asia to Oceania and began to spread to the Americas and Europe in the late 1700s. China is an important distribution and cultivation centre of *L. indica*, and has been cultivated there for >1600 years, reaching a prosperous period in the Tang Dynasty [2]. The reason for the popularity of crape myrtle is that it blooms at a time when most trees are not blooming, and it is covered with blooms that will last for months during the hottest part of the summer [1]. In addition to its advantages of unique beauty and aesthetic value, it can resist pollution, absorb harmful gases and dust, and serve as an important landscape plant.

As early as the middle of the 18th century, crape myrtle was introduced into the southeast of the USA through England. By the early 20th century, it had been widely planted on the east and west coasts of the USA [3]. In the 1960s, *Lagerstroemia fauriei*, native to Japan, was introduced to America and crossed with *Lagerstroemia indica*. Hybrids of the two species generally produced excellent offspring. Zhang investigated and collected genetic resources in the *Lagerstroemia* genus and cultivars in China for the first time [4]. To date, more than 200 hybrid cultivars with diversified plant architectures, different colours, colourful leaves, and strong disease resistance have been successfully bred [2, 5, 6]. In terms of plant architecture, phenotypic and anatomical observations of internodes revealed significant positive correlations between plant height, internode length, and cell number, and internode length was positively regulated by gibberellin [7, 8]. Differentially expressed genes (DEGs) and quantitative trait loci (QTLs) related to the regulation of dwarfism traits in crape myrtle were identified by transcriptomics and QTL mapping [7, 9]. Although the flower colour of crape myrtle is diversified, it still lacks blue, yellow, orange, and green flowers. Flavonoids are considered to be key factors in the determination of petal colour in crape myrtle [10]. In terms of leaf colour, anthocyanins and chlorophylls are considered to be the main determinants of purple and yellow leaf colour, respectively [11, 12]. However, the molecular mechanisms underlying the formation of these traits in *L. indica* are not clear.

Over the last 20 years, genomics research in higher plants, especially in Gramineae, Brassicaceae, Orchidaceae, and Rosaceae, has made great advances [13]. With the reduction in sequencing cost, population resequencing based on high-quality genomes can yield a large amount of variation information and multiple types of molecular markers, which are very helpful in the study of population evolution and domestication and for discovering candidate genes associated with target traits based on the genome-wide association study (GWAS) technique [14, 15]. However, the absence of reference genomes for Myrtaceae species has limited our understanding of systematic genomics research. Based on high-quality genomes, new insights can be gained from the analysis of the formation and evolution of important traits. In Myrtales, except for the reports that the genomes of eucalyptus (*Eucalyptus grandis*) [16], pomegranate (*Punica granatum*) [17], water caltrop (*Trapa natans*) [18], clove (*Syzygium aromaticum*) [19], and other economic tree species have been completed, only the whole set of mangrove genomes is left to explore the evolution process of tropical coastal ecosystems [20]. Whole-genome duplication (WGD), which took place during the evolutionary history of the majority of plant species and offered the potential for new functions and species diversity, could also improve species fitness and resistance. Myrtaceae plants such as *Rhodomyrtus tomentosa*, *E. grandis* and *P. granatum* shared a WGD event from 66.58 to 95.5 million years ago (MYA).

As *L. indica* is one of the most representative plants in the *Lagerstroemia* genus, it is urgent to obtain its genome and systematically conduct functional genomics research. Karyotype analysis with 45S rDNA-FISH showed that the chromosomes of *Lagerstroemia* species are small and numerous (2*n* = 2*x* = 48), consistent with the results of flow cytometry of 10 species of *Lagerstroemia* (341.00 ± 2.00–370.00 ± 8.89 Mb) [21, 22]. Due to the lack of a reference genome, very large datasets cannot be effectively integrated and utilized, which seriously hinders research on the evolution, domestication, and molecular breeding design of crape myrtle and *Lagerstroemia* species.

Here we obtained the chromosome-level genome of *L. indica* by using PacBio and Hi-C technology, performed genome resequencing and evolutionary analysis of 73 closely related species and cultivars, constructed a high-density genetic linkage map by resequencing and QTL mapping for plant height and revealed comprehensive models of plant architecture, petal colour, and leaf colour by multi-omics. This study will provide an important platform for genetic breeding and ornamental trait improvement in *L. indica*.

## Results

### Chromosome-scale reference genome assembly of *L. indica*

**Figure 1:**
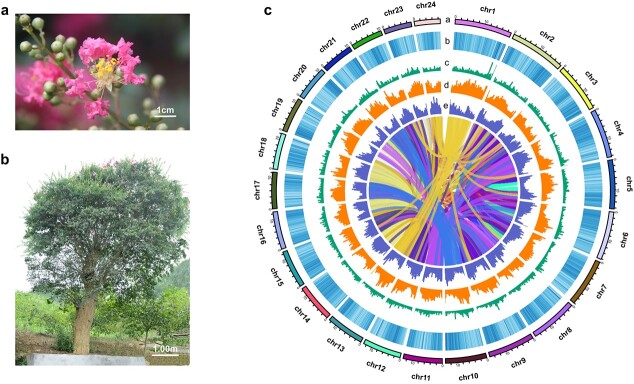
Circos display of the *L. indica* genomic features. **a** Flowers of *L. indica*. **b** The >80-year-old *L. indica* included in this study. **c** Genomic features of *L. indica.* a, assembled chromosomes; b, GC content; c, tandem repeat density; d, LTRs; e, gene density. The coloured lines in the centre of the circle represent the synteny relationship of gene blocks.

A diploid (2*n* = 2*x* = 48) of *L. indica* was used for whole-genome sequencing and chromosome-level assembly with PacBio sequencing and Hi-C technologies, respectively. We obtained 100× coverage of PacBio long-read sequencing data (33.15 Gb) and 112× coverage of Hi-C paired-end reads (37.1 Gb). The complete genome assembly size of *L. indica* was 329.14 Mb with a scaffold N50 of 13.85 Mb. The genome was assembled into 24 chromosomes, and the percentage of sequences anchored to chromosomes was 99.97%. Detailed genome assembly statistics and the chromosome-scale scaffold length range are shown in Fig. 1, Table 1, and Supplementary Data Table S1.

In order to verify the assembled chromosome-level genome's integrity and accuracy, we completed BUSCO analysis to assess the completeness of the assembly with the embryophyta_odb9 dataset. In total, 92.3% complete BUSCOs were found in *L. indica*, indicating a relatively complete and high-quality genome (Supplementary Data Table S2). The heat map of chromosomal interactions showed that the strength of interactions within the same chromosome in the diagram was significantly greater than that between chromosomes, and the boundary of chromosomes was more obvious, which indicated that the effect of assembly was ideal (Supplementary Data Fig. S1).

### Genome annotation

Overall, ~138.62 Mb of genome sequences in *L. indica* were identified as repetitive elements by the repeat annotation processes mentioned above and accounted for ~42.19% of the whole genome. The detailed prediction resources and classification of TEs are listed in Supplementary Data Fig. S2, Supplementary Data Tables S3 and S4. We identified 33 608 genes in *L. indica*, with an average coding sequence (CDS) length of 1.4 kb (Supplementary Data Table S5, Supplementary Data Fig. S3), and the BUSCO evaluation of the annotated protein sequences was 93.4%. A total of 31 487 genes were functionally annotated in *L. indica*, accounting for 93.69% of all predicted genes (Supplementary Data Table S6). The non-coding RNAs (miRNAs, tRNAs, rRNAs and snRNAs) were also annotated and are presented in Supplementary Data Table S7.

### Comparative genomic and evolutionary analysis

The 17 species contained a total of 33 875 gene families, of which 16 126 gene families (29 357 genes) were found in *L. indica* (Supplementary Data Table S8, Supplementary Data Fig. S4). Clustering of gene families from four species (*L. indica*, *P. granatum*, *Arabidopsis thaliana* and *Carica papaya*) revealed that 9675 genes were common to these species, whereas 3572 genes were unique to *L. indica* (Supplementary Data Fig. S5).

**Table 1:** Summary of the genome assembly.

Feature	Value
Estimate of genome size	329.14 Mb
Repetitive elements	138.62 Mb (42.19%)
Total number of contigs	1140
Total length of contigs	328.59 Mb
Average length of contigs	288.2 kb
N50 length of contigs	513.85 kb
N90 length of contigs	148.92 kb
Maximum length of contigs	2.42 Mb
Minimum length of contigs	11.47 kb
Total number of scaffolds	32
Total length of scaffolds	329.14 Mb
Gap number of scaffolds	554 000
Average length of scaffolds	10.29 Mb
N50 length of scaffolds	13.85 Mb
N90 length of scaffolds	10.86 Mb
Maximum length of scaffolds	19.18 Mb
Minimum length of scaffolds	114.73 kb
GC content	38.86%
Complete BUSCOs	92.30%
Number of genes	33 608
Average length of CDS	1404.02 bp
Number of miRNAs	157
Number of tRNAs	571
Number of rRNAs	327
Number of snRNAs	225

**Figure 2:**
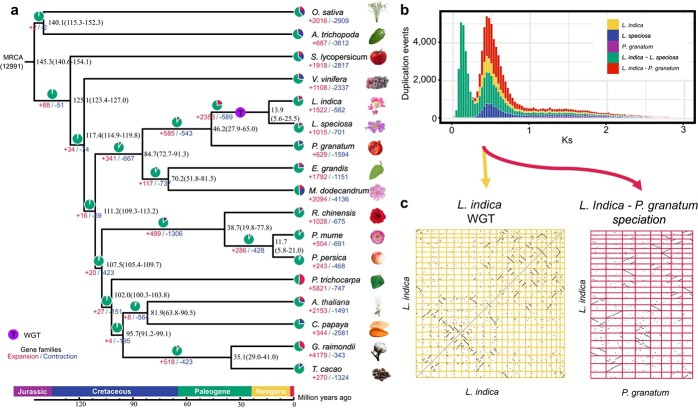
Comparative genomic and evolutionary analysis of *L. indica*. **a** Pie charts show the proportions of gene families that underwent expansion or contraction. Inferred divergence dates (in millions of years) are indicated in black at each node. Circles with "T" represent WGT events. **b***K*_s_ distributions. According to the *K*_s_ peak values in descending order, they are labeled as *L. indica*–*P. granatum* orthologues, *L. indica*–*L. speciosa* orthologues, *L. indica* paralogues, *L. speciosa* paralogues, and *P. granatum* paralogues. **c** Dot plots of paralogues from the *L. indica* WGT and speciation of *L. indica*–*P. granatum*.

Using single-copy gene families, the Bayes method was employed to construct phylogenetic trees (Fig. 2a). The phylogenetic tree showed that *L. indica* was clustered with *Lagerstroemia speciosa* as one branch and subsequently with *P. granatum*. These three species of the Lythraceae, in turn, were clustered with *E. grandis* and *Melastoma dodecandrum* in the same order as Myrtales. Based on the molecular clock, nucleotide substitution rates and *K*_s_ values, the divergence time between *L. indica* and *L. speciosa* was 10.36 million years ago (MYA) (13.9 MYA according to the molecular clock), whereas that between *L. indica* and *P. granatum* was 37 MYA (46.2 MYA according to the molecular clock) (Fig. 2a and b).

To identify polyploidization events in *L. indica*, we performed collinear alignments within the *L. indica* species and between *L. speciosa* and *P. granatum*. As shown in Fig. 2c, each chromosome of *L. indica* has a significant collinear relationship with the other two chromosomes, e.g. chr1 with chr13 and chr24. This strongly indicates a recent whole-genome triplication (WGT) event in *L. indica* [23]. Each chromosome of *P. granatum* has obvious collinearity with three *L. indica* chromosomes, such as chr8 of *P. granatum* with chr1, chr13, and chr24 of *L. indica*, which also verifies this conclusion. Each chromosome of *L. indica* has obvious collinearity with the three chromosomes of *L. speciosa* (Supplementary Data Fig. S6). The collinearity between *L. indica* and other species, such as *P. trichocarpa* and *Prunus persica*, was not significant (Supplementary Data Fig. S6), which is consistent with the conclusion of current species taxonomy. To further analyse polyploidization time across species, we calculated the rate of synonymous substitutions per site (*K*_s_) between pairs of paralogous genes within the species *L. indica*, *L. speciosa*, *P. granatum*, *M. dodecandrum*, and *Vitis vinifera*. The WGT event of *L. indica* occurred at *K*_s_ = 0.44, from which we inferred that the time of WGT was ~35.5 MYA (Fig. 2b). This occurred almost simultaneously with the polyploidization event of *L. speciosa*, which was between the two polyploidization events in *M. dodecandrum* and much later than that in *P. granatum* (Supplementary Data Fig. S7). The WGT event allowed clear expansion of the gene family after divergence from *P. granatum*; 2353 gene families were expanded and 589 were contracted (Fig. 2a). After the divergence of *L. indica* from *L. speciosa*, there was expansion of 1522 gene families and contraction of 582 gene families. *Punica granatum*, which did not undergo polyploidization during this period, instead showed expansion of 629 gene families and contraction of 1594 gene families after their differentiation from *L. indica* and *L. speciosa* [17]. Therefore, WGT events in *L. indica* played an important role in its gene family expansion. Gene ontology (GO) enrichment analysis was performed on 2353 expanded gene families with *P* ≤ .01, and they were found to be mainly enriched in adenyl ribonucleotide binding and ribonucleotide binding (Supplementary Data Fig. S8). We detected three genes subject to positive selection by calculating *K*_a_/*K*_s_ values, including chr24_0445, chr3_0239, and chr11_0492. Chr3_0239 is a homologue of *ATG4*, a cell autophagy-related gene that is closely related to plant growth, development, and stress response [24].

In brief, we suggest that a previously undescribed independent triplication event occurred after the differentiation of *L. indica* from *P. granatum*, after which *L. indica* was further differentiated from *L. speciosa*. The differentiation of *L. indica* and *P. granatum* and the triploidization of *L. indica* occurred intensively in the period of 35.5–37 MYA, i.e. during the Oligocene of the Palaeogene.

### Resequencing and population structure analysis

**Figure 3:**
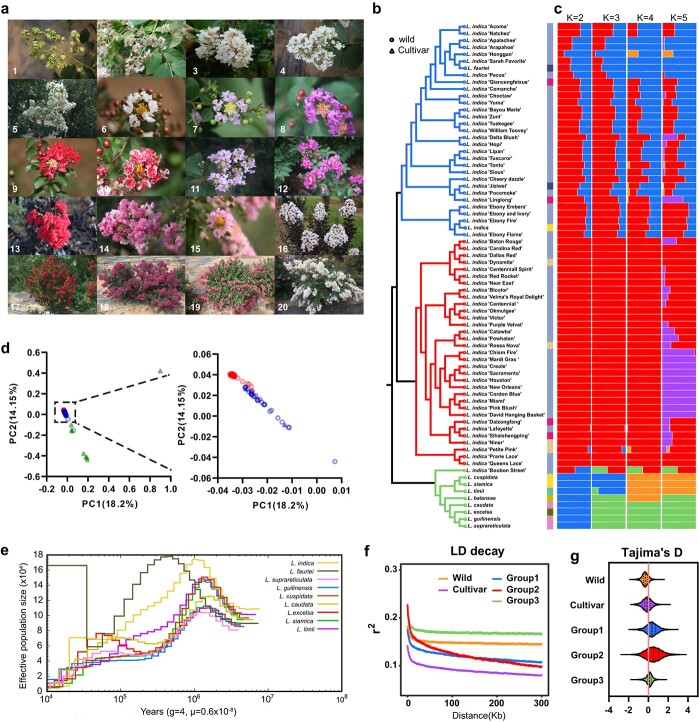
Population structure of 73 domesticated and wild *Lagerstroemia*. **a** Images of several representative accessions (1, *L. excelsa*; 2, *L. suprareticulata*; 3, *L. caudata*; 4, *L. fauriei*; 5, ‘Sarah’s Favorite’; 6, ‘Niner’; 7, ‘Yuma’; 8, ‘Catawba’; 9, ‘Dallas Red’; 10, ‘Prairie Lace’; 11, ‘Arapahoe’; 12, ‘Centennial’; 13, ‘Ebony Fire’; 14, ‘Bicolor’; 15, ‘Choctaw’; 16, ‘Ebony and Ivory’; 17, ‘Dynamite’; 18, ‘Velma's Royal Delight’; 19, ‘Pocomoke’; 20, ‘Acoma’). **b** Neighbour-joining phylogenetic tree of the 73 accessions. Differently coloured blocks represent different places of origin. Open triangles represent wild accessions and open circles represent cultivars. **c** Bayesian model-based clustering of the 73 accessions with the number of ancestral groups (*K*) from 2 to 5. The *x* axis quantifies cluster membership and the *y* axis lists the different accessions. Each colour represents one putative ancestral background, and the *x*-axis quantifies ancestry membership. **d** PCA of the 73 accessions. Dot colours correspond to colours of the clades in Fig. 1a. Open circles represent cultivars, triangles represent wild accessions. **e** Population demographic history of nine wild species of *Lagerstroemia*. **f** LD decay in different subpopulations. Groups 1, 2, and 3 are the three subpopulations delimited by the phylogenetic tree in panel **b**. **g** Tajima’s *D* estimation in different subpopulations.

A total of 73 accessions, including *L. indica*, 63 cultivars, and 9 closely related species of *L. indica*, were chosen for genome sequencing (Fig. 3a). A total of 406.24 Gb of high-quality cleaned sequences were produced by resequencing the 73 accession genomes, with an average of 5.56 Gb (13.77×) per accession. (Supplementary Data Table S9). A final set of 1 702 584 single-nucleotide polymorphisms (SNPs) were identified after the reads were aligned to the crape myrtle genome (Supplementary Data Table S10).

By creating a neighbour-joining (NJ) phylogenetic tree using SNPs, we first looked at the phylogeny between wild and *Lagerstroemia* cultivars. The closely related species of crape myrtle from various provinces in China are classified into one clade, including *L. excelsa*, *L. siamica*, *L. guilinensis*, *L. suprareticulata*, *L. balansae*, and *L. limii* (Fig. 3b). The rest of the accessions are classified into two clades, mainly including modern crape myrtle cultivars from the USA, France, Japan, and China. The last two clades contain two wild accessions, *L. indica* and *L. fauriei*, which are the main parents used for breeding modern crape myrtle cultivars.

We estimated ancestry proportions for each accession using admixed models and a Bayesian clustering algorithm to better understand the evolutionary history of crape myrtle (Fig. 3c). The best model for these 73 accessions was three populations (*K* = 3), according to the cross-validation (CV) error analysis (Supplementary Data Fig. S9). For *K* = 2, clade 3 (closely related species) showed a distinct ancestral background from clade 2, whereas clade 1 had both ancestral structures. For *K* = 3, clade 3 was further divided into two subpopulations with different ancestral backgrounds. When *K* = 4, clade 3 was divided into two subpopulations. The closely related species from Hainan, Fujian, and Yunnan provinces had a common ancestral origin, while those from Guangxi and Guizhou were grouped together. Clade 3 showed an ancestral background unrelated to that of clades 1 and 2, indicating their high diversity and farther distance from cultivars. When *K* = 5, clade 2 appeared to have two subpopulations of ancestral background. The results indicate that the ancestral origins of the three clades differ significantly. Clade 1 shares a common ancestral origin with clade 3, while the ancestral background of clade 2 differs significantly from the other two ancestral backgrounds. This indicates that the breeding parents of clade 1 may include parents from China, while the breeding parents of clade 2 may have originated in other areas.

A principal component analysis (PCA) illustrated a pattern similar to that of the phylogenetic tree. The dispersion of closely related species away from other accessions suggests distant affinities within the group. The apparent dispersion within this group indicates its rich polymorphism. In agreement with the phylogenetic analysis, the cultivars and wild accessions of *L. indica* and *L. fauriei* were clustered together. The species material was densely clustered together (Fig. 3d).

To evaluate the population size fluctuations of nine wild species, we used the pairwise sequential Markovian coalescent (PSMC) method. (Fig. 3e). We found a peak in population size ~1 MYA, followed by a downwards trend. *Lagerstroemia fauriei* clearly diverged from other species, especially with the rapid rise in population size ~30 000 years ago, probably due to its geographical isolation from others.

The linkage disequilibrium (LD) of crape myrtle displayed a half-decay value within 5 kb for the wild accessions but extended to 9 kb in the cultivars (Fig. 3f). To investigate potential selection patterns, we estimated the Tajima's *D* values of various subpopulations. As depicted in Fig. 3g, we noticed a gradual increase in negative Tajima's *D* values as accessions changed from wild to cultivar status, indicating increased positive selection during varietal development. Comparing cultivar accessions with wild accessions, we found that the nucleotide diversity was higher in the cultivar accessions (Supplementary Data Fig. S10). The cultivars had more diversity than the wild accessions, as evidenced by the LD decay and values.

### Construction of a high-density genetic linkage map

**Figure 4:**
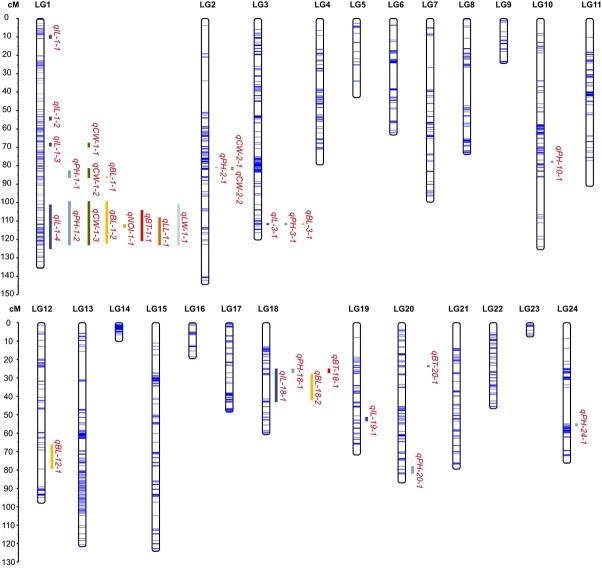
High-density genetic map of crape myrtle and QTL mapping of plant architecture traits. The QTLs are named as follows: q-trait abbreviation-linkage group number-linkage group sequential number. Trait abbreviations: PH, plant height; CW, crown width; BL, branch length; IL, internode length; NOI, number of internodes; BT, branch thickness; LL, leaf length; LW, leaf width. QTLs for the same trait are represented by lines of the same colour.

An *F*_1_ population of 361 progenies was constructed with *L. fauriei* (female) and *L. indica* ‘Pocomoke’ (male) as parents. Whole-genome resequencing was performed on both parents and their progenies. In total, 13.52 (40.09×) and 10.41 Gb (31.64×) of clean reads were generated for *L. fauriei* and ‘Pocomoke’, respectively. Moreover, 1234.33 Gb of cleaned sequences were generated for 361 individuals from *F*_1_ populations (average 9.1×) with high quality (Q20 ≥ 96.31%, Q30 ≥ 90.13%; Supplementary Data Table S11). A total of 11 884 117 polymorphic loci were obtained through polymorphism development between parents (Supplementary Data Tables S12 and S13). After genotyping, the progeny markers were screened, and 27 839 subsequent markers were obtained. After removing the severely unlinked markers, the markers were divided into 24 linkage groups (LGs) according to chromosomes. To construct the genetic linkage map, 5660 SNP markers were eventually acquired. The map consisted of 24 LGs and covered 1853 cM with an average distance of 0.33 cM (Fig. 4, Supplementary Data Tables S14 and S15).

### Analysis of plant architecture traits and QTL mapping in *L. indica*

**Figure 5:**
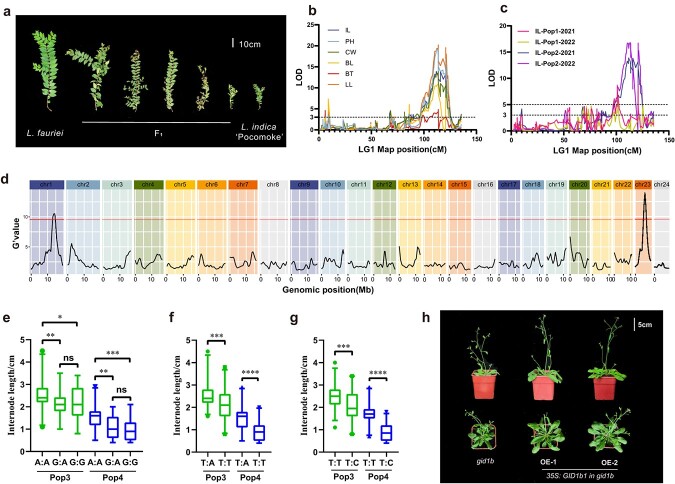
QTL mapping of internode length by genetic linkage mapping and BSA. **a** Branch phenotypes of *L. fauriei* (non-dwarf), ‘Pocomoke’ (dwarf) and their hybrid *F*_1_. **b** QTL mapping on LG1 for several plant architecture traits, including IL, PH, CW, BL, BT, and LL. **c** QTL mapping of IL in four environments. Pop1, 139 *F*_1_ individuals obtained in 2011. Pop2, 222 *F*_1_ individuals obtained in 2020. **d** Distribution of internode length G′ values on each chromosome of *L. indica* based on BSA. **e**–**g** Phenotypes of multigeneration population IL under different genotypes of A016052 (**e**), A016053 (**f**), and A016054 (**g**). Pop3, 91 BC_1_ individuals obtained in 2014 and 53 *F*_2_ individuals obtained in 2014. Pop4, 48 *F*_1_ individuals obtained in 2018 and 80 *F*_1_ individuals obtained in 2019. Asterisks indicate significant differences, according to Student’s *t* test (^*^P < .05; ^**^P < .01; ^***^P < .001; ^****^P < .0001). **h** Plant height and crown width and features of *LfiGID1b1* transgenic *A. thaliana* seedlings.

Plant architecture is a broad and complex synthetic trait. Here, 15 phenotypic trait indicators (Supplementary Data Table S16) were used to decompose plant architecture. We measured 15 phenotypic traits of 361 progenies (138 plants 11 years old and 223 plants 2 years old) for 2 years. (Fig. 5a). The coefficient of variation of the plant architecture traits in this population was 30.00–141.00%. The genetic variation in plant height (PH), crown width (CW), leaf length (LL), leaf width (LW), branch length (BL), number of branches (NOB), branch series (BS), and number of branches/axillary buds (B/AB) was >40%. Among these traits, PH, CW, IL, BL, branch thickness (BT), and other traits related to growth were significantly and positively correlated (Supplementary Data Fig. S11). Further examination of the phenotype frequency distributions revealed that PH, CW, BL, internode length (IL), BA, POA, BT, number of internodes (NOI), number of axillary buds (NOAB), NOB, B/AB, LL, and LW fitted or approximately fitted a normal distribution and were suitable for QTL analysis (Supplementary Data Figs S12 and S13).

QTL analysis was performed on the map using the above 13 traits. QTL intervals were first filtered by LOD > GW and LOD > 3, within which regions co-localizing in more than two environments were selected. Finally, we mapped 33 intervals explaining 7.6–38.3% of the phenotypic variation (Fig. 4, Supplementary Data Table S17). Among the four environments, traits including PH, CW, BL, and IL co-localized to the interval 99.01–125.33 cM of LG1 with LOD >16.49 and PVE >32.3 (Pop2-IL-2021) (Fig. 5b), which indicates that there is a main effect interval that simultaneously regulates growth-related traits such as PH, IL, and LL. IL is a key trait determining the PH and BL of *L. indica*, which is easy to measure, little affected by the environment, and stable in the four environments (Supplementary Data Fig. S14). By QTL analysis of the internode length data from the four environments, we mapped the main-effect interval on LG1 (Fig. 5c). The interval was mapped to 13 234 008–16 923 969 bp on the reference genome.

**Figure 6:**
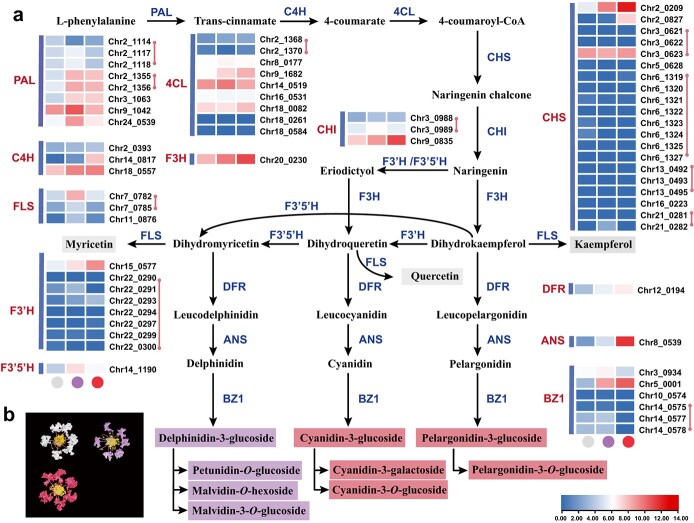
Biosynthesis pathways of two kinds of flavonoids, anthocyanins, and flavonols. **a** Gene expression profiles of different petal colours. The left side of the heat map shows the corresponding enzymes, and the right side shows the gene IDs within the genome. Genes identified in tandem clusters are marked with a red line. PAL, phenylalanine ammonia-lyase; C4H, cinnamate-4-hydroxylase; 4CL, 4-coumarate CoA ligase 4; CHS, chalcone synthase; CHI, chalcone isomerase; F3H flavanone 3-hydroxylase; F3′H, flavonoid 3′-hydroxylase; F3′5′H, flavonoid 3′5′-hydroxylase; FLS, flavonol synthase; DFR, dihydroflavonol 4-reductase; ANS, anthocyanidin synthase; F3oGT, flavonoide-3-*O*-glucosyltransferase; BZ1, bronze-1. **b** Three differently coloured flowers of *L. indica* used for RNA-seq.

### Bulk segregant analysis-based mapping of internode length

To obtain more reliable results, we localized the IL in combination with BSA. We extracted 124.42 Gb of resequencing data from the two parents and the individuals with extreme internode length (Fig. 5e). The average read depth was 11.97× in ‘Pocomoke’, 10.52× in *L. fauriei*, 135.06× in the short IL pool and 131.46× in the long IL pool (Supplementary Data Table S18). Among the 869 113 polymorphic markers identified, 822 623 were selected for SNP index analysis, each of which had a read depth of >7 and was biallelic between ‘Pocomoke’ and *L. fauriei*. We analysed the data with QTLseqr and calculated the G′ value plotted to the genome position. Two genomic regions (chr1: 12527119-15407629 and chr23: 5064585–6624838) were discovered to have a G′ value above the cutoff with a 99% significance level (Fig. 5d). BSA and the genetic linkage map were jointly mapped to the interval of 13 234 008–15 407 629 bp on chr1, which may be the main effective interval for controlling internode length. In order to further verify the accuracy and versatility of the localization interval, we further developed KASP markers and tested them in BC_1_, *F*_2_, and other *F*_1_ populations. We have designed a total of nine KASP markers, of which seven were successfully developed and five were genotyped (Fig. 5e–g, Supplementary Data Fig. S15). The final results showed that the genotyping of A016052, A016053, and A016054 markers was significantly correlated with phenotype, confirming the accuracy of QTL mapping. The A016052 markers showed that IL_A:A_ > IL_G:A_ (*P*_pop1_ = .0046, *P*_pop2_ = .0089) and IL_A:A_ > IL_G:G_ (*P*_pop1_ = .4231, *P*_pop2_ = .0004), while there was no significant difference in internode length between the G:A and G:G genotypes (Fig. 5e). The A016053 marker showed that IL_T:A_ > IL_T:T_ (*P*_pop1_ = .0008, *P*_pop2_ < .0001, Fig. 5f). The A016054 marker showed that IL_T:T_ > IL_T:C_ (*P*_pop1_ = .0004, *P*_pop2_ < .0001, Fig. 5g). There were 166 genes in this interval; chr1_1120 (*LfiCDL15*) associated with cyclin, chr1_1200 (*LfiCRG98*) associated with lignin, auxin, and response to environmental stresses, and chr1_1206 (*LfiGID1b1*) is a gibberellin receptor gene (Supplementary Data Table S19). To investigate the function of *LfiGID1b1*, it was transformed into the *gid1b* mutant of *A. thaliana* (Fig. 5h, Supplementary Data Fig. S16). Overexpression of *LfiGID1b1* resulted in a 26.67% increase in plant height (Supplementary Data Fig. S17a), 31.75% increase in crown width (Supplementary Data Fig. S17b), and 20.4% increase in leaf length in seedlings (Supplementary Data Fig. S17c), compared with the control. These findings suggest that *LfiGID1b1* may be a key regulator of plant height and crown width in *L. indica*.

**Figure 7:**
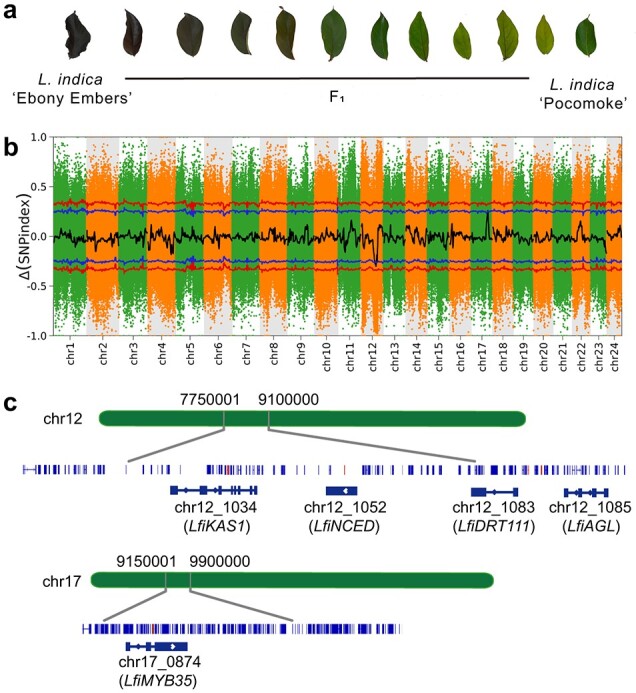
Location and map-based cloning of loci for purple leaves in crape myrtle. **a** Progenies of the crape myrtle segregating population with different leaf colours. **b** ΔSNP index values used for the association analysis. The *x*-axis and *y*-axis indicate the 24 *L. indica* chromosomes and the ΔSNP index, respectively. The black line represents the fitted ΔSNP index. The outer and inner lines indicate the threshold for association with leaf colour at the 99 and 95% confidence intervals, respectively. **c** Candidate genes and their locations on chromosomes. Details include the location of the mapped segment on the chromosome, distribution of genes within the mapped interval, and genes closely related to leaf colour.

### Flavonoid pathways contribute to crape myrtle flowers

Transcriptome sequencing of 12 samples taken from *L. indica* flowers with white, red, and purple flowers yielded a total of 309.54 Gb of clean reads, with an average of 25.795 Gb of clean reads per sample (Fig. 6b, Supplementary Data Table S20). Pairwise alignment of these three sets of transcriptomes revealed DEGs (Supplementary Data Table S21). DEGs in various colour samples were found by analysing transcripts gleaned from the transcriptome and categorizing them based on their FPKM values. There were 7184 DEGs in red versus white (4204 downregulated, 2980 upregulated), 6087 DEGs in red versus purple (3673 downregulated, 2414 upregulated), and 5983 DEGs in white versus purple (2949 downregulated, 3034 upregulated) (Supplementary Data Fig. S18). Enrichment analysis of these DEGs revealed that, in addition to being enriched in flavonoid pathways, there were also a large number of DEGs involved in metabolic pathways and biosynthesis of secondary metabolites (Supplementary Data Fig. S19).

Through genome and transcriptome sequencing data, we reconstructed the metabolic pathway of flower coloration, which included 64 enzymatic genes associated with anthocyanin and flavonol biosynthesis (Fig. 6a). Flavonoid synthesis pathways include coloured anthocyanin pigments and colourless flavonols. Twelve important gene families in this pathway were identified, among which the *PAL* (8), *4CL* (9), *CHS* (20), and *F3′H* (8) gene families had a large number of members (Fig. 6a). In addition, we found that members of the *PAL*, *4CL*, *CHS*, *CHI*, *FLS*, *F3*′*H*, and *BZ1* genes were replicated, with tandem duplication observed. The gene expression levels of the same gene clusters were similar, such as chr2_1114, chr2_1117, and chr2_1118 in *PAL*. Different gene clusters of the same gene family often show uncorrelated expression. Among these tandemly repeated gene clusters, chr2_1355, chr2_1356, and chr9_1042 in *PAL*, chr18_0557 in *C4H*, chr8_0177, chr9_1682, chr14_0519, chr16_0531, and chr18_0082 in *4CL*, chr2_0209 and chr3_0623 in *CHS*, chr9_0835 in *CHI*, chr15_0577 in *F3'H*, chr7_0782 in *FLS*, chr3_0934, and chr5_0001 in *BZ1* were significantly highly expressed. The expression levels of these genes were also significantly different among different petal colours. The expression of these genes in white flowers was lowest among the three petal colours, while it was generally higher in red flowers. The formation of white flowers is the result of low expression of many enzymes in the flavonoid pathway, not the low expression of one enzyme. After naringenin formation, the expression levels of *F3′H*, *DFR*, *ANS* and *BZ1* in red flowers were higher than those in purple flowers, while the expression levels of *F3'5'H* and *FLS* in purple flowers were higher than those in red flowers.

### Bulk segregant analysis-based mapping of leaf colour genes

In total, 75.83 Gb of resequencing data were generated from two parents and two extreme individual pools (green leaf and purple leaf) (Fig. 7a). The average read depth was 15.08× in ‘Pocomoke’, 57.92× in *L. indica* ‘Ebony Embers’, 17.59× in the green pool, and 21.74× in the purple pool (Supplementary Data Table S22). A total of 3 613 881 polymorphic markers were selected for SNP index analysis, each of which had a read depth of >15 and was biallelic between ‘Pocomoke’ and ‘Ebony Embers’. Δ(SNP index) was calculated as the difference between the SNP index values of the two pools and plotted against the positions on the reference genome. There were two regions where the mean line exceeded the threshold line in the results, which were chr12: 7 750 001–9 100 000 bp and chr17: 9 150 001–9 900 000 bp (Fig. 7b). Within these regions, we identified five candidate genes based on gene function: chr12_1052, chr12_1083, chr12_1085, chr12_1034, and chr17_0874 (Fig. 7c). Chr12_1052 is annotated to the carotenoid synthesis pathway and is a homologue of *NCED*. Chr12_1083, chr12_1085, and chr12_1034 were annotated to chloroplast-related pathways and were homologous to *DRT111*, *AGL*, and *KAS1*, respectively. Chr17_0874 was annotated to the anthocyanin pathway and is a homologue of *MYB35* (Supplementary Data Table S23).

## Discussion

*Lagerstroemia indica* has high ornamental value due to its colourful, long-lasting panicles of flowers, as well as its elegant and beautiful trunk revealed by the peeling bark and the rich colour of the older stems [25]. Here we describe the genome of *L. indica* for the first time and assemble it to the chromosome level. The construction of this genome lays a foundation for research on the genetics, evolution, germplasm material population structure, and molecular breeding of *L. indica*. WGDs are widespread in the plant kingdom and are significant for species diversity, the origin of new gene functions, and the enhancement of stress resistance [26]. In addition to the gamma (γ) WGT event in the ancestor of core eudicots, recent WGD events have also occurred in different species [27–29], and these duplications are often closely related to changes in the environment. The chromosome numbers of *R. tomentosa*, *E. grandis*, and *P. granatum* were 11, 8, and 11, respectively. The significantly greater number of chromosomes in *L. indica* than in the former three species is due to a single independent WGT event at 35.5 MYA in *L. indica*. Shared WGD events in Myrtaceae were not apparent in *L. indica*, mainly because the new duplication event would eliminate traces of previous WGD events to some extent. The period 23.03–33.9 MYA falls within the Palaeogene-Oligocene, and a single large-scale species extinction event occurred during the early Oligocene [30]. We detected the differentiation of *L. indica* and *P. granatum*, and the triploidization events of *L. indica* occurred intensively during this period, which implies that the evolution of *L. indica* is related to global climate change (Supplementary Data Fig. S20). Only a very small number of genes would have been under positive selection, and by *K*_a_/*K*_s_ analysis we identified three genes under positive selection (*K*_a_/*K*_s_ ≫ 1). Among them, *ATG4* is associated with plant growth, development, and stress response, indicating that its non-synonymous mutations confer stronger environmental adaptation to *L. indica* [31].

In this study, we performed deep resequencing of 73 closely related species and cultivars of crape myrtle. Population structure analysis shows us the history and current status of breeding modern cultivars of crape myrtle [32]. Among the wild species, only *L. indica* from China and *L. fauriei* from Japan were clustered with modern crape myrtle cultivars, demonstrating that they are the main parents for modern crape myrtle cultivars. Asia is the main origin centre of *L. indica*, and China, Europe, and the USA are the main domestication centres [33]. *Lagerstroemia fauriei,* native to Yakushima, Japan, was used by American breeders, including Egolf [34], to improve crape myrtle cultivars because of its excellent resistance to powdery mildew. Its close relationship with crape myrtle may also be another important reason for its application in breeding. Many modern cultivars are of *L. fauriei* origin, and this breeding process was verified in the present structural analysis (Fig. 3). Wild closely related species from the warm, humid regions of China are not clustered with the cultivars, indicating that these species are minimally involved in the breeding of modern crape myrtle cultivars. These species that have not been used in modern crape myrtle breeding may contain new genes that provide richer genetic resources for resistance, flower type, petal colour, and plant architecture and are of great value for future crape myrtle breeding.

In crops and horticultural plants, people tend to breed by artificial selection that preserves advantageous variants [14, 32]. Convergent selection of the WD40 protein occurred even in two different species, maize and rice [35]. Therefore, in most crops and horticultural plants, breeding is a process of reduced genetic diversity. However, the opposite is true in ornamental plants such as crape myrtle, where breeding increases the genetic diversity of cultivars. This phenomenon occurs because the breeding direction of ornamental plants is chosen to enrich diversity, whereas that in crops decreases diversity.

Plant architecture has long been the focus of plant research because of its important influence on yield, harvest, ornamental, and other characteristics [36]. Remarkable achievements have been made in research on plant architecture in herbaceous plants represented by Gramineae [37–39]. Woody plants have many unique characteristics, such as continuous growth, rhythmic growth, and seasonal regulation of branching, and have important economic value; however, research on woody plant architecture is very limited [40, 41]. Plant architecture is a complex comprehensive trait [42]. Previous studies have shown that the plant architecture traits of *L. indica* are regulated by multiple genes [43]. High-density genetic linkage maps can be used to simultaneously localize multiple genes. We constructed a genetic linkage map of *L. indica*, which has the largest mapping population, the largest number of upper markers, and the smallest average distance between markers, providing a research reference for the study of woody plant architecture. We conducted QTL mapping of 13 plant architecture traits through two years of phenotypic trait data. Thirty-three QTL intervals were obtained, which laid a foundation for future studies of *L. indica* plant architecture.

In this study we used a combination of genetic linkage mapping and BSA to map the IL trait and localized it to a major-effect regulatory locus in the interval from chr1 13 234 008 to 15 407 629 bp, indicating the accuracy and effectiveness of the synergistic use of the two methods. By developing KASP markers, we obtained genotyping and phenotypic co-segregation of three markers, A016052, A016053, and A016054, and verified the accuracy of QTL mapping at the population level. Chr1_1120 (*LfiCDL15*) was annotated to be associated with cyclin, which can affect cell number and in turn regulate IL [44]. Chr1_1200 (*LfiCRG98*) was annotated to be associated with auxin and response to environmental stresses. Chr1_1206 (*LfiGID1b1*) is a homologue of *GID1b*, a gibberellin receptor gene. Gibberellins are important hormones that regulate plant architecture. The *SD* [45] and *RHT* [46] genes, which are involved in gibberellin signalling, were responsible for the Green Revolution in cereal crops. In woody plants, a mutation in *PeGID1c* in peach leads to dwarfism [47]. In a previous study, gibberellins were found to have a significant effect on IL in crape myrtle [7]. In this study, we transferred the *LfiGID1b1* gene into *A. thaliana* and observed increases in plant height, crown width, and leaf length, indicating that *LfiGID1b1* also plays a role in regulating plant growth in *L. indica*. The flowers of *L. indica* have diversified colours, and different kinds and contents of anthocyanins ultimately determine flower colours [29, 48]. According to the determination of metabolites, flavonoids are the main chromogenic substance driving the petal colour of *L. indica* [10, 49]. As in many plants, DEGs in the flavonoid pathway have an important effect on flower colour formation [28, 50]. In this study, we detected the expression of genes involved in the anthocyanin synthesis pathway and flavonol synthesis pathway through transcriptome analysis. The occurrence of copies of the same gene family on different chromosomes has been strongly associated with WGT events, including *PAL*, *CHS*, and *F3′H*. We speculate that WGT events can provide more genetic materials for anthocyanin production to make petals more diverse and colourful. The expression levels of different copies of the upstream *PAL* gene are generally high. However, significant differences in expression levels between different copies exist in some downstream genes, and only a few highly expressed genes may play a critical role, such as chr2_0209 and chr3_0623 in *CHS*. Flower colour breeding has always been an important direction in ornamental breeding, and yellow flowers have always been one of the directions pursued by *L. indica* breeders. Researchers measured the petal colour metabolites of the *Heimia* genus with yellow flowers and *L. indica* and found that the flavones and flavonols in the *Heimia* genus were significantly more abundant than those in *L. indica*, which was speculated to be the reason for the yellow flowers [10]. *FNS* is an important enzyme gene for flavone synthesis. *FNS* is absent in many plants [51], including *A. thaliana*, and we found no copies of *FNS* in the reference genomes of *L. indica*, indicating that deletion of the *FNS* gene may block the synthesis of flavone in *L. indica*, thus leading to the lack of yellow flowers in this species.

The type, concentration, and distribution of leaf pigments determine leaf colour [52, 53]. A leaf's chromogenic pigments are mainly chlorophylls, carotenoids, and anthocyanins. Anthocyanins mainly exhibit a range of colours from red to blue and play a dominant role in the colour rendering of red (purple) leaves [54, 55]. Previous studies on the metabolome and transcriptome of the leaves of purple *L. indica* have suggested that anthocyanins are key factors in the formation of purple leaves in *L. indica* [12]. In this study, we mapped leaf colour-related QTLs to chr12 and chr17 using BSA in *L. indica* for the first time. Leaf colour formation has been well characterized in many plants and is mainly regulated by three key pigments, chlorophylls, carotenoids, and anthocyanins [56, 57]. Chlorophylls render leaves green, carotenoids render leaves yellow, and anthocyanins are pigments that render leaves purple [53]. In the QTL regions, five genes associated with these pigments were considered candidates. Chr12_1034 (*LfiKAS1*), chr12_1083 (*LfiDRT111*), and Lin_chr12_1085 (*LfiAGL*) are associated with chlorophyll, and it has been shown that the chlorophyll content determines yellow leaf formation in crape myrtle [11]. *NCED* (chr12_1052 homologous gene) is often considered to be associated with adversity stress, but it has recently been shown that its role in β-carotenoid pathways is as a key gene in the regulation of apricot flesh colour formation [58]. Chr17_0874 (*LfiMYB35*) belongs to the MYB family, which is one of the most critical transcription factor families for purple leaf formation [12].

In summary, based on the completion of high-quality genome sequencing of *L. indica*, we not only clarified the evolutionary status of *Lagerstroemia* but also provided a reference for revealing the mechanism underlying important ornamental traits.

## Materials and methods

### Plant materials

Fresh young leaves used for genome sequencing and Hi-C analysis were sampled from an *L. indica* individual >80 years old in Baokang County, Hubei Province, China. Seventy-three domesticated and wild *Lagerstroemia* samples were collected from the nursery of the National Engineering Research Center for Floriculture in Beijing. The nine closely related species were sampled from different provinces of China and Yakushima, Japan. Sixty-three cultivars were sampled from the USA, China, France, and Japan. Fresh leaves of 73 samples were subjected to genome resequencing. An *F*_1_ population with 361 progenies was obtained using *L. fauriei* as the female and *L. indica* ‘Pocomoke’ as the male for genetic linkage mapping analysis. Fresh leaves were collected from both parents and offspring for genome resequencing. The populations used for KASP marker development included 48 *F*_1_ individuals obtained in 2018, 80 *F*_1_ individuals obtained in 2019, 91 BC_1_ individuals obtained in 2014, and 53 *F*_2_ individuals obtained in 2014. For RNA-seq, >20 petals of flowers from *L. indica* ‘Acoma’ (white flowers), ‘Dallas Red’ (red flowers), and ‘Apalachee’ (purple flowers) were collected at 6 a.m. in August. Four biological replicates of each sample were included. For BSA-seq, 97 *F*_1_ progenies were obtained by crossing ‘Pocomoke’ (green leaf) as the female parent with ‘Ebony Embers’ (purple leaf) as the male parent. Fresh leaves were collected from parents, 32 green-leaf progenies, and 32 purple-leaf progenies.

### Genomic sequencing and resequencing

Purified DNA samples were used in the construction of third-generation DNA libraries, Hi-C libraries, and regular short-read WGS libraries. The third-generation DNA libraries were sequenced on the PacBio platform. WGS libraries and Hi-C libraries were all sequenced on the BGISEQ-500 platform at the Qingdao Huada Gene Research Institute and were used in the polishing and scaffolding of draft assemblies.

Seventy-three *Lagerstroemia*-related species and cultivars and 361 genetic mapping populations of crape myrtle were sequenced using Illumina™ PE150. After processing, adaptor and low-quality sequences were taken out of the unique reads. The cleaned unique reads were aligned to the *L. indica* reference genome using BWA (parameters: mem -t 4 -k 32 -M -R) [59], and only uniquely mapped reads were retained. We used SAMTOOLS (parameter: rmdup) [60] for the detection of population SNPs. To obtain high-quality SNPs, we utilized Bayesian models to detect polymorphic loci within the population. The results of the SNP assay were annotated using ANNOVAR [61].

### *De novo* genome assembly

A draft contig-level genome was first assembled using PacBio sequencing data and CANU (v.1.8, parameters: MhapSensitivity = high corMinCoverage = 4 minReadLength = 1500 minOverlapLength = 500 corOutCoverage = 200 ‘batOptions=-dg 3 -db 3 -dr 1 -ca 500 -cp 50’). The raw result was then subjected to two rounds of polishing with WGS data in Pilon (v.1.23). Lastly, Hi-C read pairs were mapped to the polished assembly with HiC-Pro [62], and Juicer [63] and 3D-DNA [64] were used to correct and locate the final positions of all contigs to construct a chromosome-level genome assembly.

### Repeat identification

RepeatMasker (http://repeatmasker.org/RMDownload.html) and RepeatProteinMask were employed to detect tandem and interspersed repeats by identifying repeat elements as homology predictions based on RepBase (http://www.girinst.org/repbase). This step was crucial prior to using RepeatModeler (http://repeatmasker.org/RepeatModeler/) for structure analysis and function annotation. Moreover, the LTR FINDER [65] and TRF [66] tools were employed to predict repeat elements *de novo*, using the features of the repeat sequences.

### Genome annotation

Evidence was collected from three resources for genome structure annotation analysis: *de novo* prediction, homologue alignment, and transcript annotation. Augustus [67], Genscan [68] and GlimmerHMM [20] were used to conduct *de novo* prediction, and several representative sequenced plants were used to finish homologue alignment, including *Arabidopsis thaliana*, *Brassica napus*, *Camelina sativa*, *Cucumis melo*, *E. grandis*, *Eutrema salsugineum*, *Gossypium raimondii*, *P. granatum*, *Raphanus sativus*, and *Theobroma cacao*. Our genome assembly was mapped to all these reference species using BLAT [69], and the protein-coding area was predicted by GeneWise [70]. The sequence data of RNA from all kinds of sequenced tissues were combined and mapped to our assembly using HISAT [71], and then transcripts were annotated using StringTie [72] and TransDecoder. All these annotation results were then combined by EVM (v.1.1.1) to obtain the final genome structure.

Functional annotation of genes predicted in the assembly data was carried out using the KEGG, SwissProt, and TrEMBL (https://www.uniprot.org/) databases through BLASTp (E-value = 1E−5) analysis. Gene function at the domain level was predicted using both GO [73] and InterPro.

Identification of non-coding RNAs (ncRNAs) is a crucial component of genome annotation. Due to sequence conservation, plant ribosomal RNA (rRNA) data were used as a reference and mapped to the *L. indica* genome using BLASTn (E-value = 1E−5). Transfer RNA (tRNA) prediction was performed using tRNAscan-SE v.1.3.1 [74], and small nuclear RNAs (snRNAs) and microRNAs (miRNAs) were discovered using the Rfam database (snRNAs).

### Genome duplication and intergenomic comparison

OrthoMCL [75] was utilized to cluster gene families from a total of 17 species, including *A. thaliana*, *C. papaya*, *P. trichocarpa*, *Rosa chinensis*, *Prunus mume*, *Prunus persica*, *E. grandis*, *Melastoma dodecandrum*, *V. vinifera*, *Solanum lycopersicum*, *Oryza sativa*, *G. raimondii*, *T. cacao*, *Amborella trichopoda*, *L. indica*, *L. speciosa*, and *P. granatum*, for a total of 17 species. To build the phylogenetic tree, gene family clustering analysis was performed on these species. A Venn diagram of gene family clustering in four species (*L. indica*, *L. speciosa*, *P. granatum*, and *P. mume*) was generated.

Phylogenetic trees were constructed using single-copy gene families. The alignment of protein sequences from single-copy gene families commenced with the use of Muscle [76]. Protein sequences were then reverse transcribed into CDSs based on the alignment results, and each aligned 4-fold degenerate site was extracted for concatenation into a supergene. Tree formation was then performed using MrBayes [77], and dendrogram files were imaged using FigTree.

MCMCTree in the PAML5 [78] package was used to estimate species divergence times with the ‘correlated molecular clock’ model and the ‘F84’ nucleic acid substitution model. The corrected divergence times were acquired via the TimeTree website (http://www.timetree.org/). Speciation events were dated to 102.0–113.8 MYA for *L. indica*–*P. mume*, 70.0–91.1 MYA for *L. speciosa–E. grandis*, 143.0–174.8 MYA for *A. thaliana*–*O. sativa*, 179.0–199.1 MYA for *A. trichopoda*–*P. trichocarpa*, 28.7–40.9 MYA for *G. raimondii*–*T. cacao*, 102.0–113.8 MYA for *C. papaya*–*R. chinensis*, 70.0–91.1 MYA for *M. dodecandrum*–*P. granatum*, and 112.4–125.0 MYA for *S. lycopersicum–V. vinifera*. Gene family expansion and contraction analyses were performed using CAFE [79] based on gene family clustering results and phylogenetic relationships among species. Enrichment analyses for KEGG and GO were performed on 1272 genes (*P* ≤ .01) in the expansion set.

Positive selection analysis was performed using CodeML [80] in PAML, with the ‘branch site’ model, using the target species vetch as the foreground branch and other species clustered by gene family as the background branch (*P* < .05).

MCScanX was used to discover collinear blocks and paralogous and orthologous gene pairs [81]. The species included in the analysis were *P. trichocarpa*, *M. dodecandrum*, *V. vinifera*, *P. persica*, *L. indica*, *L. speciosa*, and *P. granatum*. TBtools Genome Gene Dotplot [82] was employed for conducting collinearity analysis. *K*_s_ (parameter: r = 6.15 × 10^−9^) values were calculated using TBtools Simple *K*_a_/*K*_s_ Calculator and plotted using the R package ggplot [82].

### Phylogeny and population structure of *L. indica*

For phylogenetic and population structure analyses, we selected a total of 1 702 584 SNPs with a minor allele frequency (MAF) ≥1% and missing rate per site ≤10%. The distance matrix was computed using TreeBeST-1.9.2 (http://treesoft.sourceforge.net/treebest.shtml), and subsequently a phylogenetic tree was generated with the NJ algorithm based on this matrix. Bootstrap values were obtained by up to 1000 calculations. PCA used GCTA [83], taking the values of PC1 and PC2 for plotting. Population structure was analysed using Plink (https://www.cog-genomics.org/plink/). Admixture software (http://dalexander.github.io/admixture/index.html) was used to obtain population genetic structure and lineage information. PSMC (parameters: g = 4, r = 6.15E-09) [84] was employed for inferring the historical dynamics of effective population size. The squared correlation coefficient (*r*^2^) between SNPs was calculated and plotted using PopLDdecay (parameter: MaxDist = 300 kb) in order to identify and contrast the patterns of LD between different subpopulations [85]. Nucleotide diversity (*π*) (parameters: -dp6-miss0.1-maf0.01 —window-pi 100 000 —window-pi-step 10 000), genetic distance (*F*_ST_) (parameters: -dp6-miss0.1-maf0.01 -window-size 100 000), and Tajima’s *D* (parameters: -dp6-miss0.1-maf0.01.vcf —TajimaD 100 000 —keep) were calculated using VCFtools [86].

### Genetic map construction

The loci with missing parental information were filtered out, and the loci conforming to the mapping marker type of the *F*_1_ population were screened out. Then, we chose markers to cover 100% of all progenies while filtering the abnormal bases. After excluding SNPs that showed significant segregation distortion (*P* < .001), the remaining genetic markers were assigned to linkage groups using the chromosome division method. Each linkage group was sequenced by LepMap3 software using the maximum likelihood approach [87]. The Kosambi function was used to calculate the genetic distance between markers.

### Phenotypic observation and QTL mapping

A total of 13 plant architecture characteristics of the parents and *F*_1_ hybrids were investigated. The detailed measurement methods and the analysis of phenotypic traits are described in Supplementary Data Table S16. We performed QTL analysis in four environments, including phenotypic traits measured for 138 progeny of 11-year seedling age in 2021 and 2022, and 223 progeny of 2-year seedling age in 2021 and 2022. By using a permutation test (PT) (parameter: 1000) in MapQTL (https://www.kyazma.nl/index.php/MapQTL/), the LOD threshold values for each phenotype were established. MapQTL software was utilized to locate QTLs by employing the MQM algorithm. The threshold selection principle was as follows: we first considered the LOD threshold of GW corresponding to 0.95 confidence. If there was still no result, we manually lowered the threshold to 3 regardless of the PT test result. The naming rule for QTLs in this study was as follows: q-trait abbreviation-linkage group number-linkage group sequential number. For example, qIL-4-1 represents the first QTL related to IL in the fourth linkage group.

### Bulked segregation analysis of internode length

The two bulks were constructed with 50 long-internode and 50 short-internode *F*_1_-generation individuals. Resequencing data for extreme individuals were extracted from genetic linkage mapping population sequencing data. The alignment and SNP detection were performed according to the genetic linkage map construction. The G′ value index (99% quantile as the threshold) values were calculated and used for the statistical analysis of allelic variations between the two bulks using the R:QTLseqr package (https://github.com/bmansfeld/QTLseqr).

### KASP assay development

For each SNP locus, a set of three primers were designed including two forward primers and one reverse primer that was common across all loci (Supplementary Data Table S24). The final reaction volume was 3 μL, composed of 1.5 μL DNA, 0.75 μL 2× KASP master mix, 0.0417 μL primer mix, and 0.75 μL ddH_2_O. PCR amplification conditions were as follows: 95°C for 15 min, 10 cycles of 94°C for 20 s, annealing temperature range 61–55°C (decreasing by 0.6°C per cycle)/1 min, followed by 26 cycles of 94°C for 20 s and 55°C for 1 min. Then, SNPviewer2 software (LGC Genomics) (http://www.lgcgroup.com) was used to analyse KASP marker data and generate genotyping results. Multiple comparisons in one-way ANOVA were analysed by GraphPad Prism 9.

### Gene cloning and plant transformation

The CDS of *LfiGID1b1* gene was amplified by PCR with specific primers (*GID1b1*-F, 5′-ATGGCCGGGAGTAACGAAGT-3′; and *GID1b1*-R, 5′-TTAAGATTGGGAAGGACTAG-3′) and cloned into the plant expression vector pSuper1300 containing a kanamycin resistance gene. The resulting recombinant vectors were transformed into *Agrobacterium tumefaciens* (GV3101) [88] and introduced into the *A. thaliana* genome using the floral-dip method [89]. More than 10 positive transgenic *LfiGID1b1* plants were obtained. All plants were grown in an artificial climate chamber with an average temperature of 22 ± 2°C, with 16 h of light and 8 h of darkness, 100 μmol m^−2^ s^−1^ light intensity, and 65–75% relative humidity.

### Transcriptome analysis

Transcriptome libraries of differently coloured flower petals were all sequenced on the BGISEQ-500 platform. Filtering was performed using SOAPnuke [90]. Reference genome alignment was performed using HISAT. We used StringTie [72] to reconstruct the transcripts for each sample, and we then compared the integrated transcripts with the reference annotation data using Cufflinks [91], which we defined as novel transcripts. CPC [92] was utilized to predict the protein coding potential of novel transcripts, and the predicted information was further integrated with the reference gene sequence, thereby resulting in the generation of complete reference sequence data. SNP and InDel information was obtained using GATK [93], and the results were stored in VCF format. Gene alignment rates were statistically analysed using Bowtie2 [94], and gene and transcript expression levels were recalculated using RSEM [95], resulting in FPKM values. Genes with differential expression were identified using the NOIseq algorithm, and DEGs were filtered according to the default criteria of fold change ≥2 and deviation from probability value ≥0.8.

### Bulked segregation analysis of leaf colour

The two bulks were constructed with 32 green-leaf progenies and 32 purple–red-leaf progenies of *F*_1_ generation individuals. DNA was extracted, and sequencing libraries were constructed for PE 100 resequencing on the BGISEQ-500 platform. Clean reads were obtained by filtering the raw reads using SOAPnuke [90] and were aligned to the CG genome by Burrows–Wheeler alignment software [59]. The SNPs and InDels were detected and filtered using the Genome Analysis Toolkit [93]. The ΔSNP index (95% quantile as the threshold) values were calculated and used for the statistical analysis of allelic variations between the two bulks with the R:QTLseqr package (https://github.com/bmansfeld/QTLseqr).

## Supplementary Material

Web_Material_uhad146

## Data Availability

The final assembly and annotation of the *L. indica* genome, the whole-genome sequences, raw resequencing data of populations, and raw transcriptome RNA-seq data are available at the National Genomics Data Centre (NGDC) under BioProject ID PRJCA013427.
