# Supplementary material for: Genome assembly and resequencing analyses provide new insights into the evolution, domestication and ornamental traits of crape myrtle
Source: Hortic Res. 2023 Jul 21;10(9):uhad146. doi: 10.1093/hr/uhad146 (PMC10493637; doi:10.1093/hr/uhad146)
Supplement: Web_Material_uhad146 [file web_material_uhad146.zip › 20230626-Supplementary Fig. 1-20.pdf]

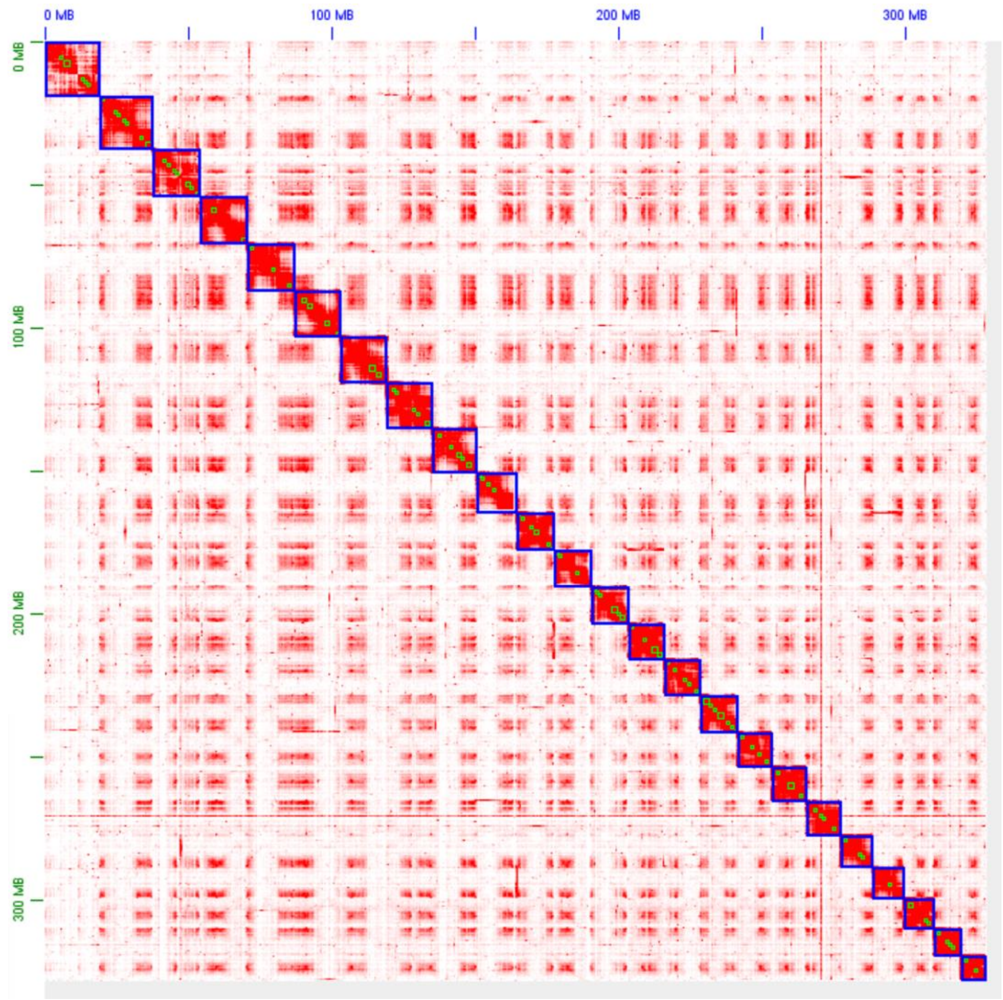

Fig. 1 Chromosomal interaction Heatmap. The darker the red color represents the stronger the interaction strength.

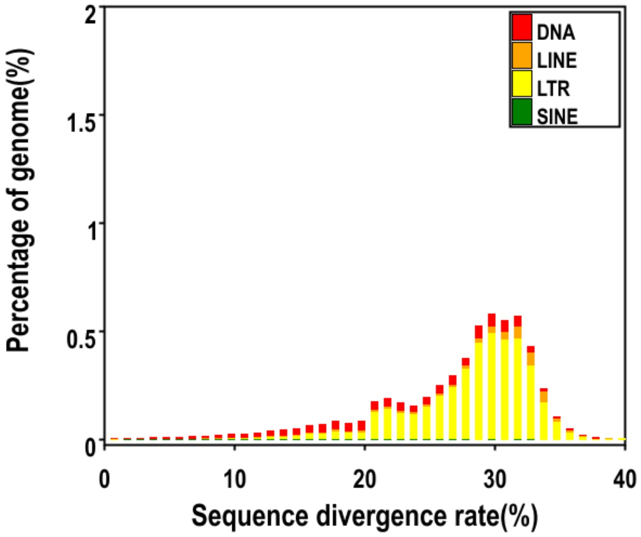

Fig. 2 TE sequence divergence profle (prediction results based on Repbase Library)

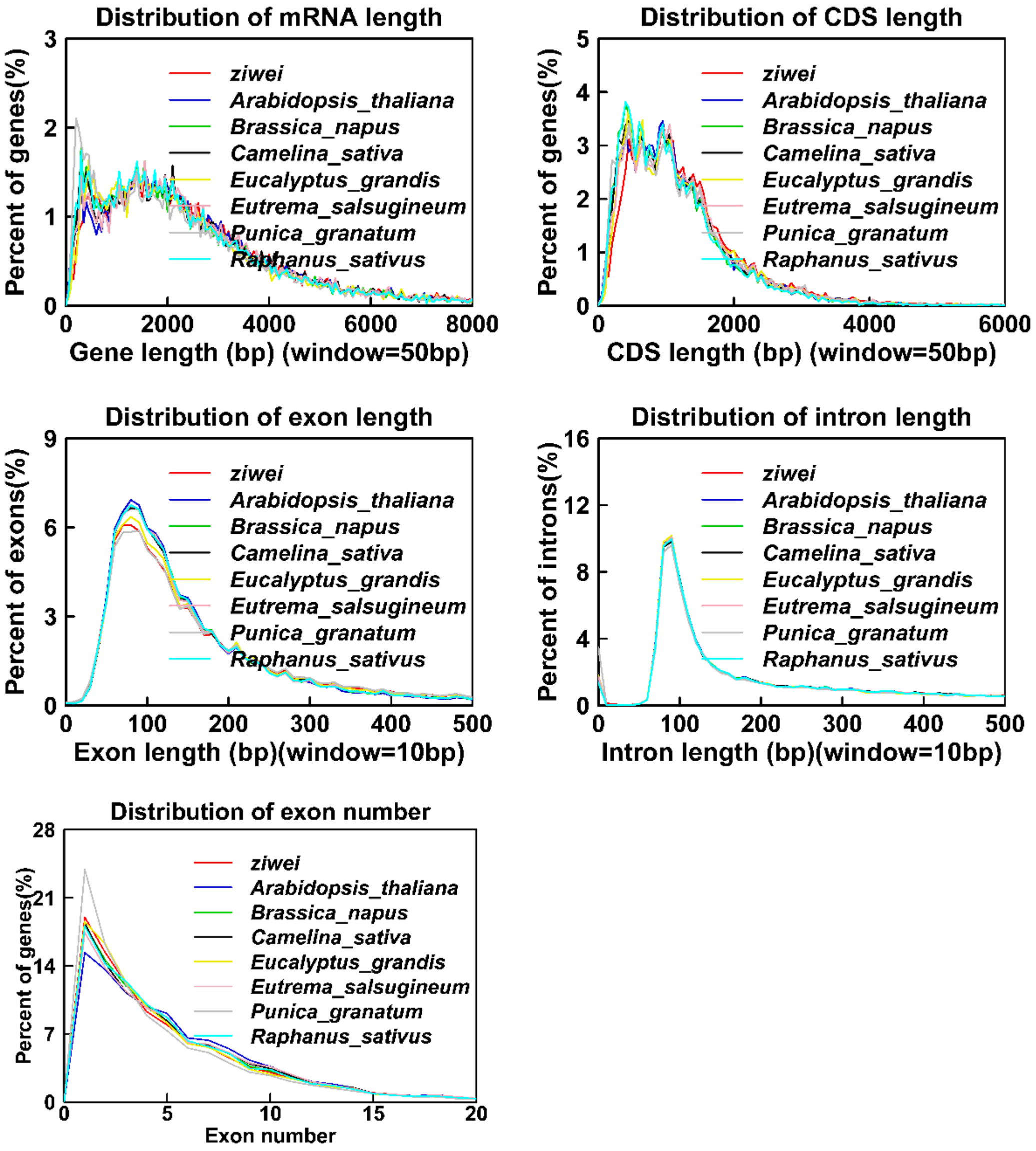

Fig. 3 Comparison of gene structure across multiple species

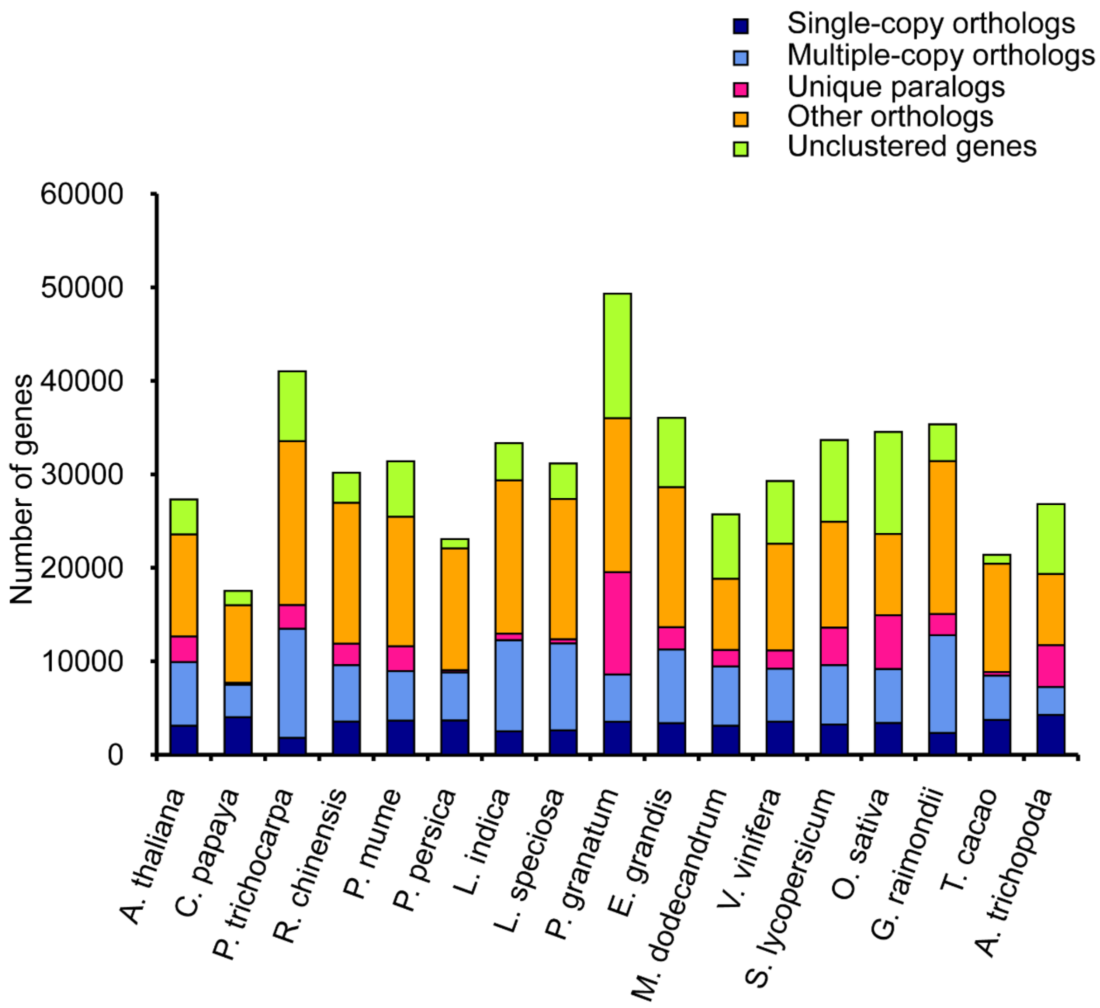

Fig. 4 Comparison of the number of homologous genes. The x-axis is species and y-axis is number of genes.

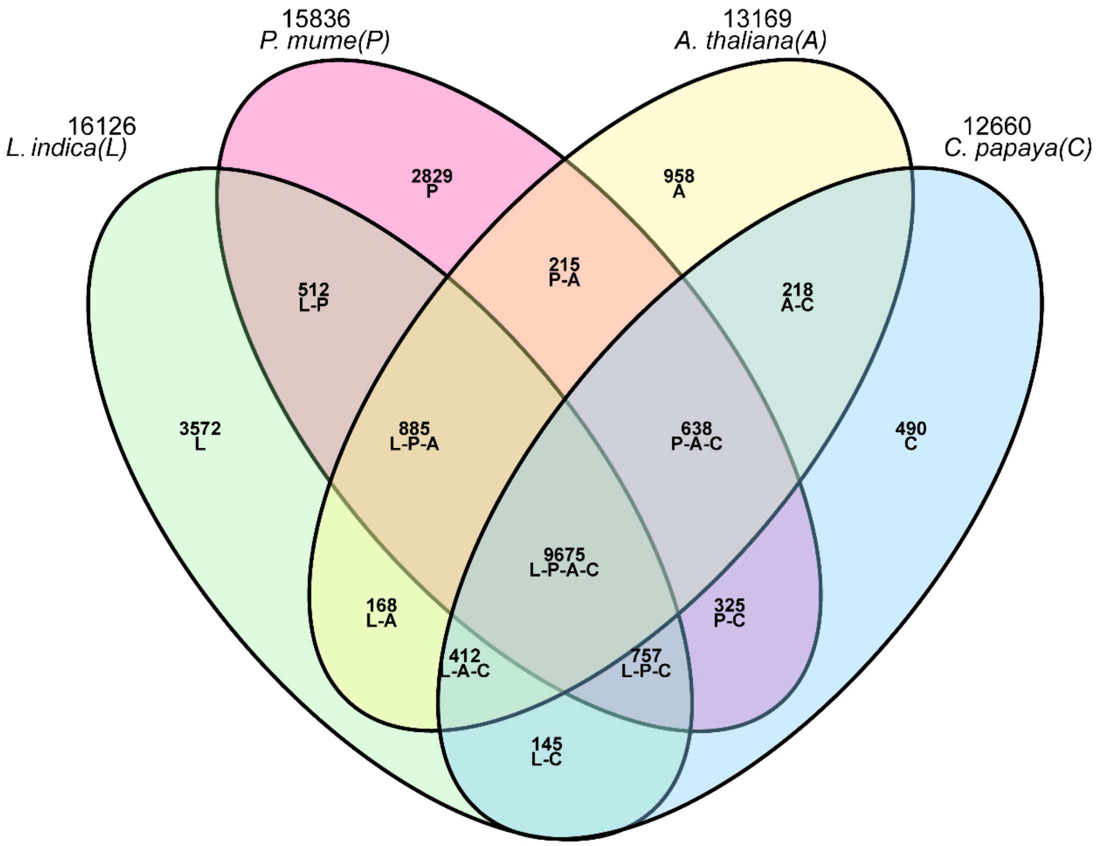

Fig. 5 The shared and unique gene families were compared among four plants (*L. indica*, *P. mume*, *A. thalinan*, *C. papaya*). Each number represents the number of gene families.

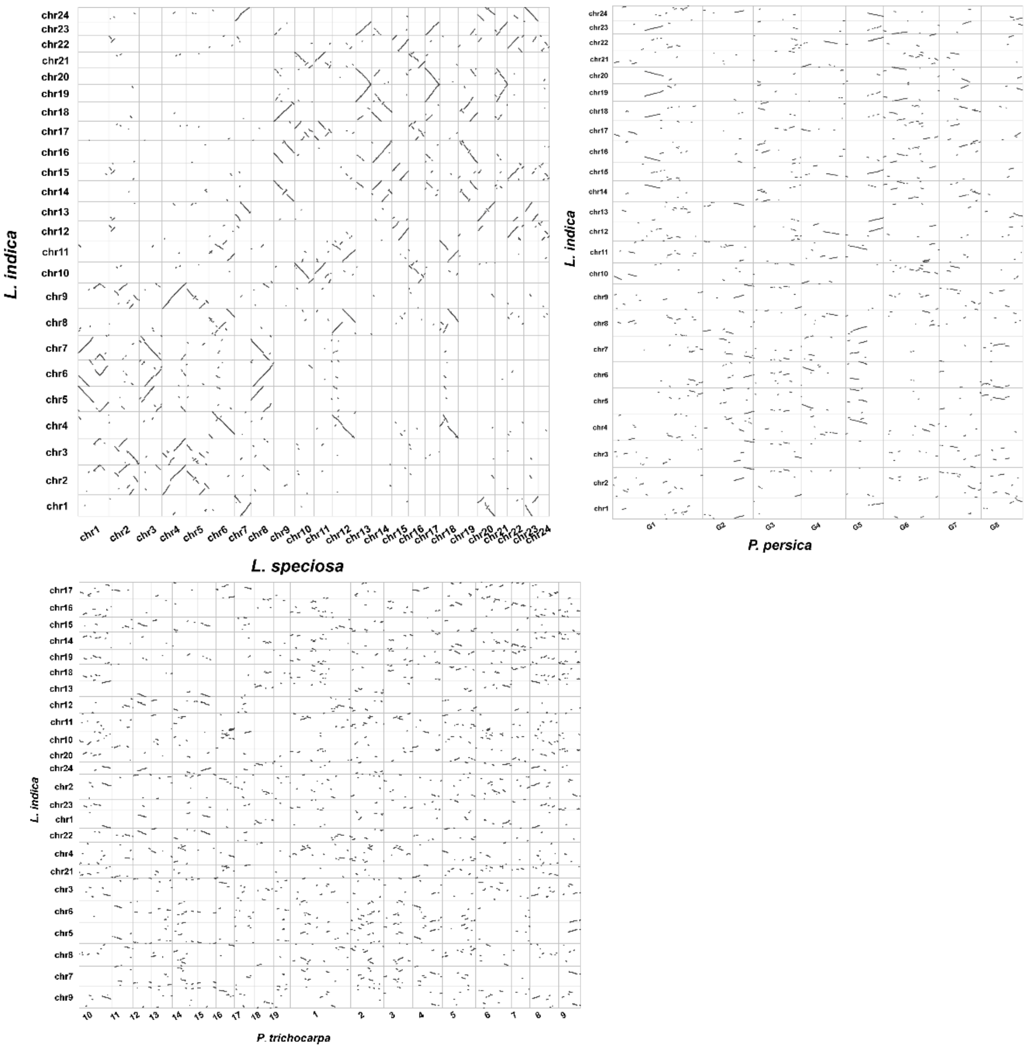

Fig. 6 Genome Dotplot of *L. speciosa*, *P. persica* and *P. trichocarpa*

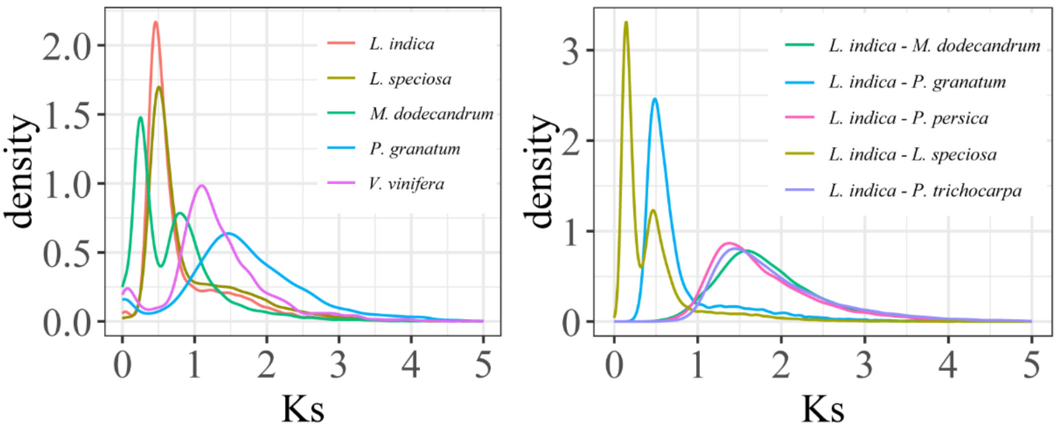

Fig. 7 Ks distribution in the left showing Ks distribution from paralogs within *L. indica*, *L. speciosa*, *M. dodecandrum*, *P. granatum*, and *V. vinifera*. Ks distribution on the right is showing Ks distribution from orthologs between *L. indica* and each of the five species (*M. dodecandrum*, *P.*

***granatum*, *P. persica*, *L. speciosa*, and *P. trichocarpa*).**

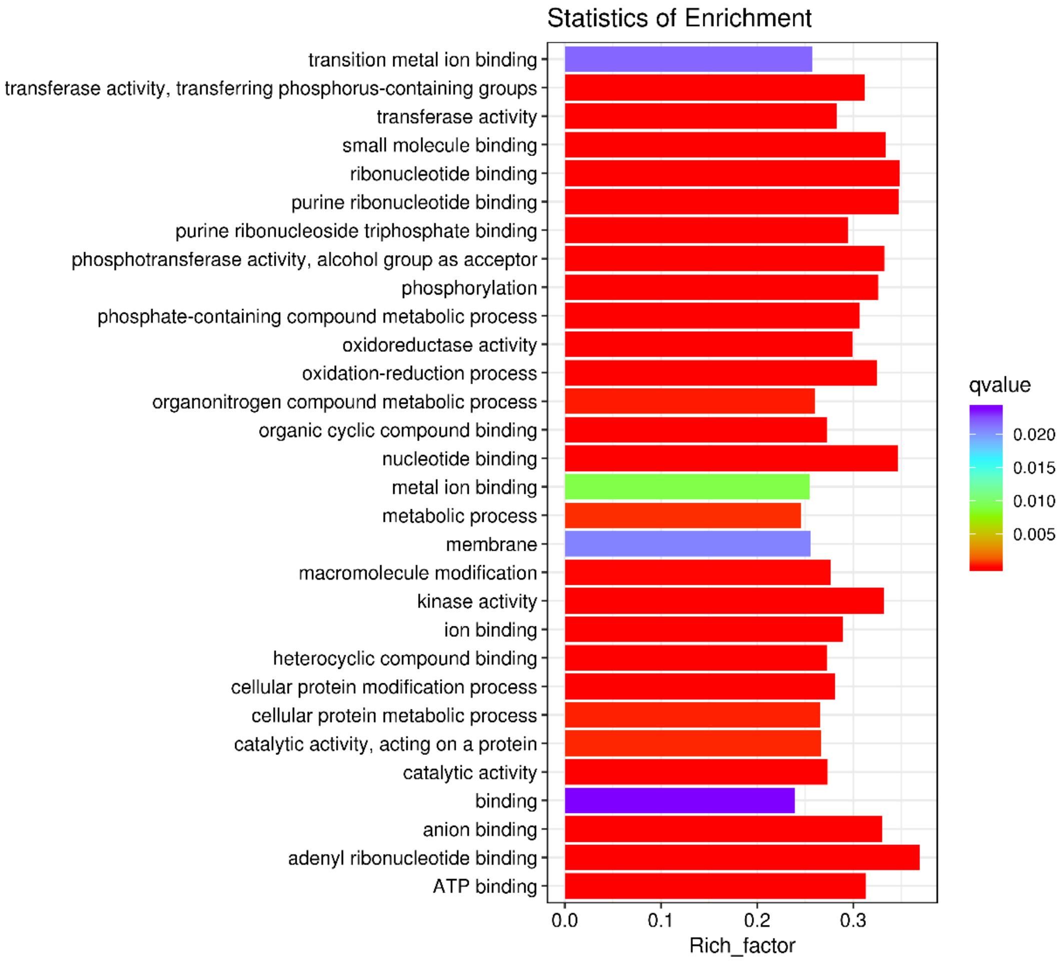

Fig. 8 Enrichment analysis of expanded gene family GO in *L. indica* after differentiation from *P. granatum*.

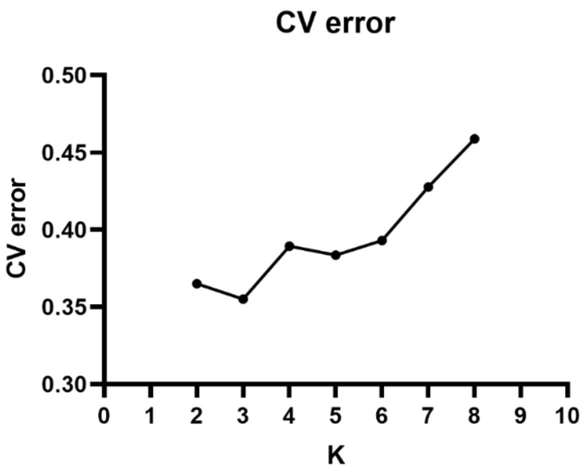

Fig. S9 73 domesticated and wild *Lagerstroemia* structure CV error.

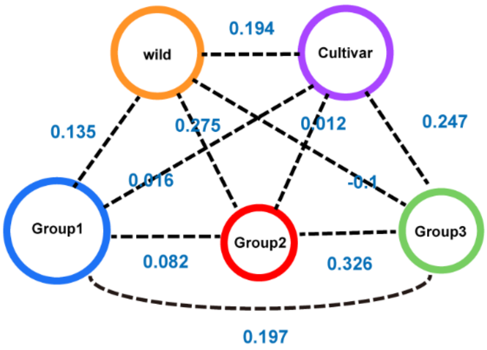

Fig. 10 Nucleotide diversity (π) and genetic differentiation (Fst) within different subpopulations calculated using the sliding-window approach (100 kb windows with 10 kb steps). The circle size represents the mean value of π in each subpopulation. The numbers between pairs of subpopulations indicate the weighted Fst values.

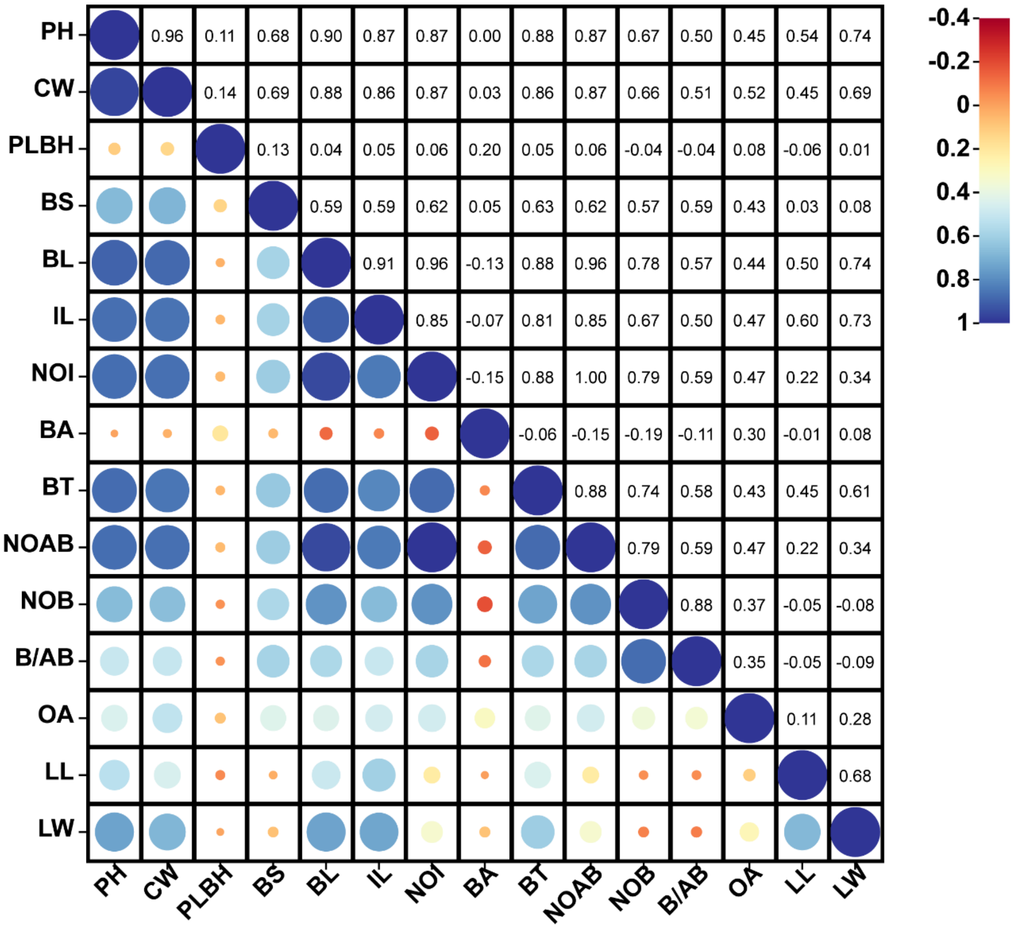

Fig. 11 Plant architecture traits correlation Heatmap. PH, Plant height; CW, Crown width; PLBH, Primary lateral branch height; BL, Branch length; IL, Internode length; NOI, Number of internode; BT, Branch thickness; BS, Branching series; BA, Branch angle; NOAB, Number of axillary bud; NOB, Number of branch; B/AB, Number of axillary bud/branch; POA, Plant opening angle; LL, Leaf length; LW, Leaf width.

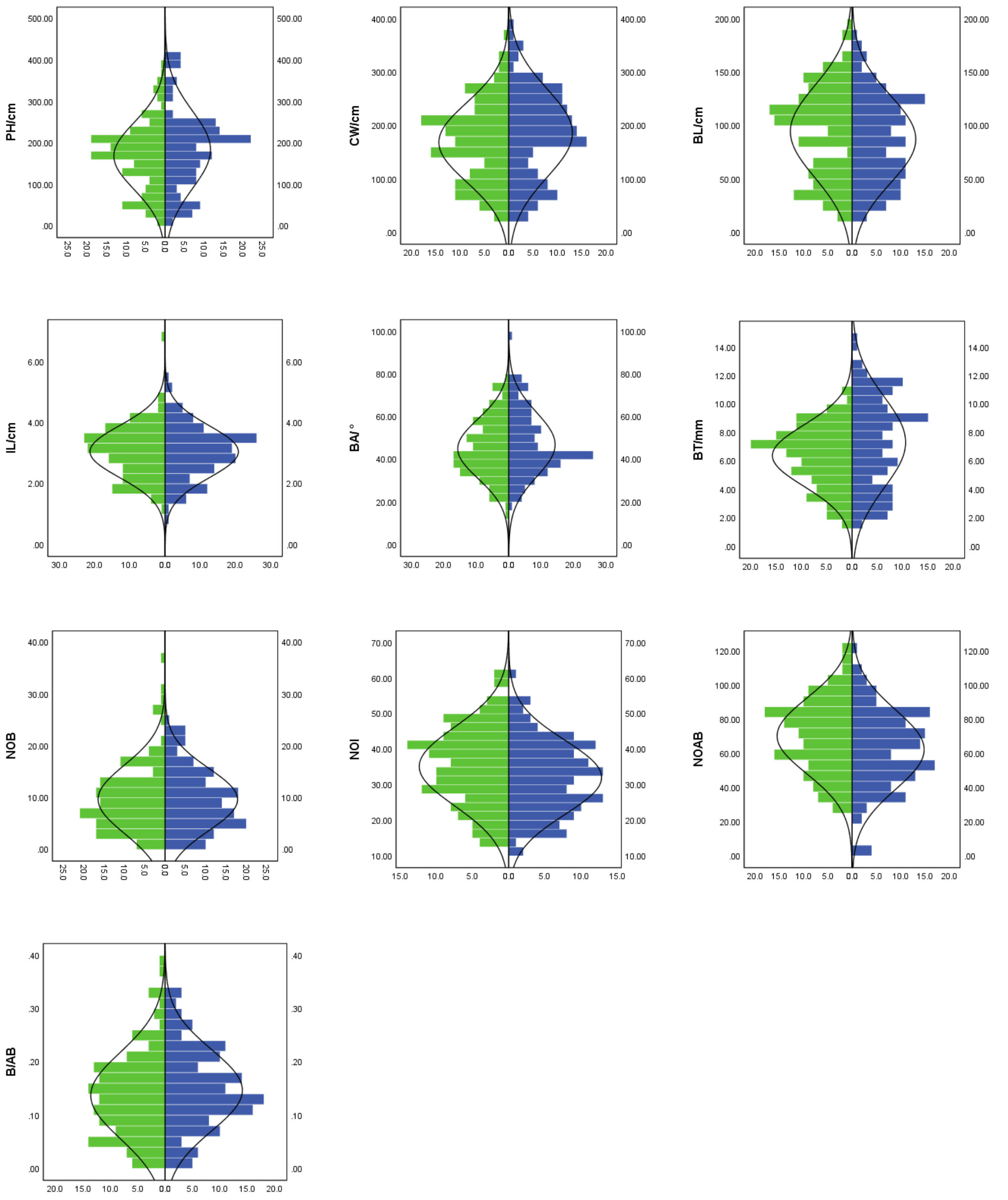

Fig. 12 Histograms of plant architecture trait frequencies in the 11-year seedling age population.

Note: Green columns represent 2021 phenotypic data and blue columns represent 2022 phenotypic data. PH, Plant height; CW, Crown width; BL, Branch length; IL, Internode length; NOI, Number of internode; BT, Branch thickness; BA, Branch angle; NOAB, Number of axillary bud; NOB, Number of branch; B/AB, Number of axillary bud/branch.

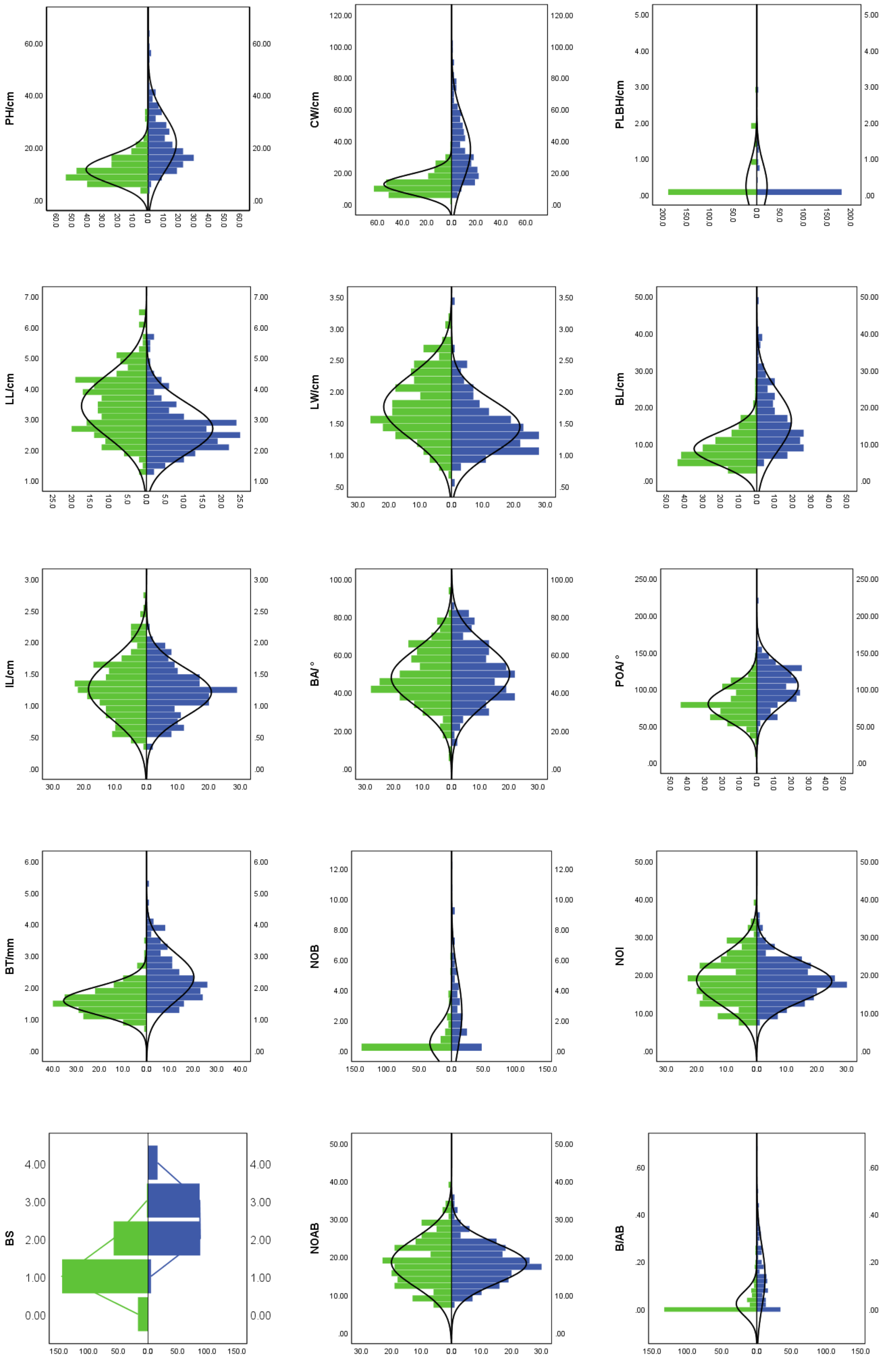

Fig. 13 Histograms of plant architecture trait frequencies in the 2-year seedling age population.

Note: Green columns represent 2021 phenotypic data and blue columns represent 2022 phenotypic

data. PH, Plant height; CW, Crown width; PLBH, Primary lateral branch height; BL, Branch length; IL, Internode length; NOI, Number of internode; BT, Branch thickness; BS, Branching series; BA, Branch angle; NOAB, Number of axillary bud; NOB, Number of branch; B/AB, Number of axillary bud/branch; POA, Plant opening angle; LL, Leaf length; LW, Leaf width.

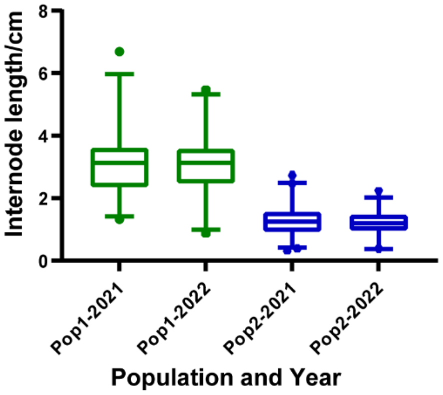

Fig. 14 Phenotype of internode length in four environments. Pop1, 139 F1 individuals obtained in 2011. Pop2, 222 F1 individuals obtained in 2020.

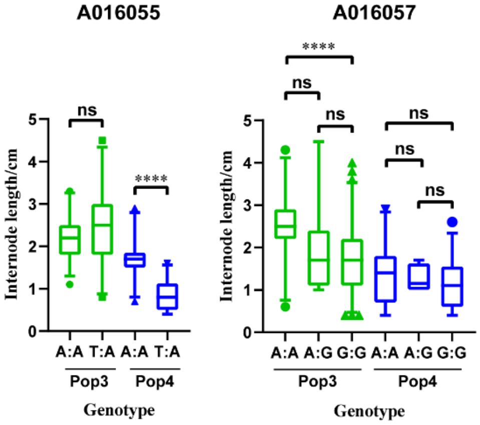

Fig. 15 Phenotypes of multi generation populations IL under different genotypes of A016055 and A016057. Pop3, 91 BC1 individuals obtained in 2014, and 53 F2 individuals obtained in 2014. Pop4, 48 F1 individuals obtained in 2018 and 80 F1 individuals obtained in 2019.

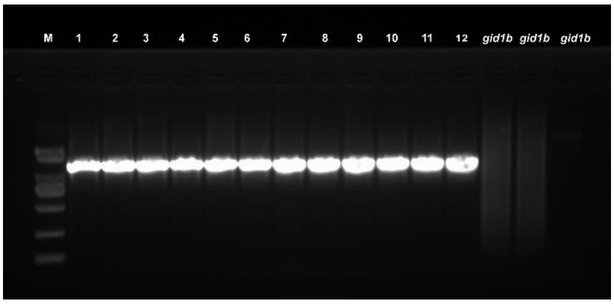

Fig. 16 PCR analysis of genomic DNA in resistant plants. M, DL2000 DNA maker. 1-12, PCR

amplification products used DNA of transgenic *A. thaliana* as PCR template. *gid1b*: PCR amplification products used DNA of mutant *gid1b*.

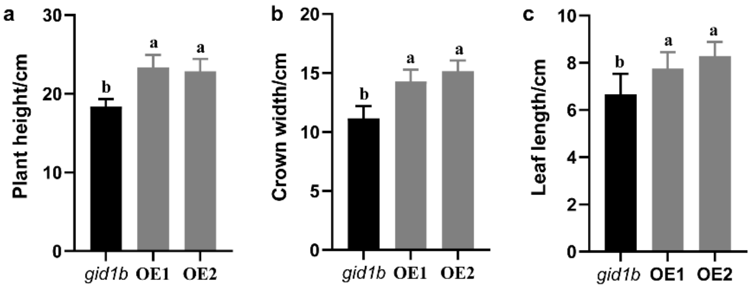

Fig. 17 Phenotypic statistics *LfiGID1b1* transgenic *A. thaliana* seedlings. a Plant height. **b** Crown width. **c** Leaf length. Different letters above the columns in the figure indicate the level of significance at $p < 0.05$.

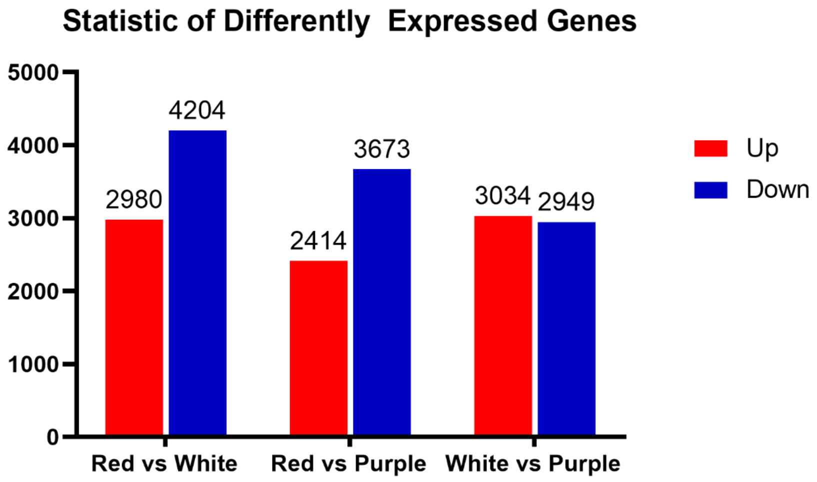

Fig. 18 Statistics of differentially expressed genes between three flower colored *L. indica* petals.

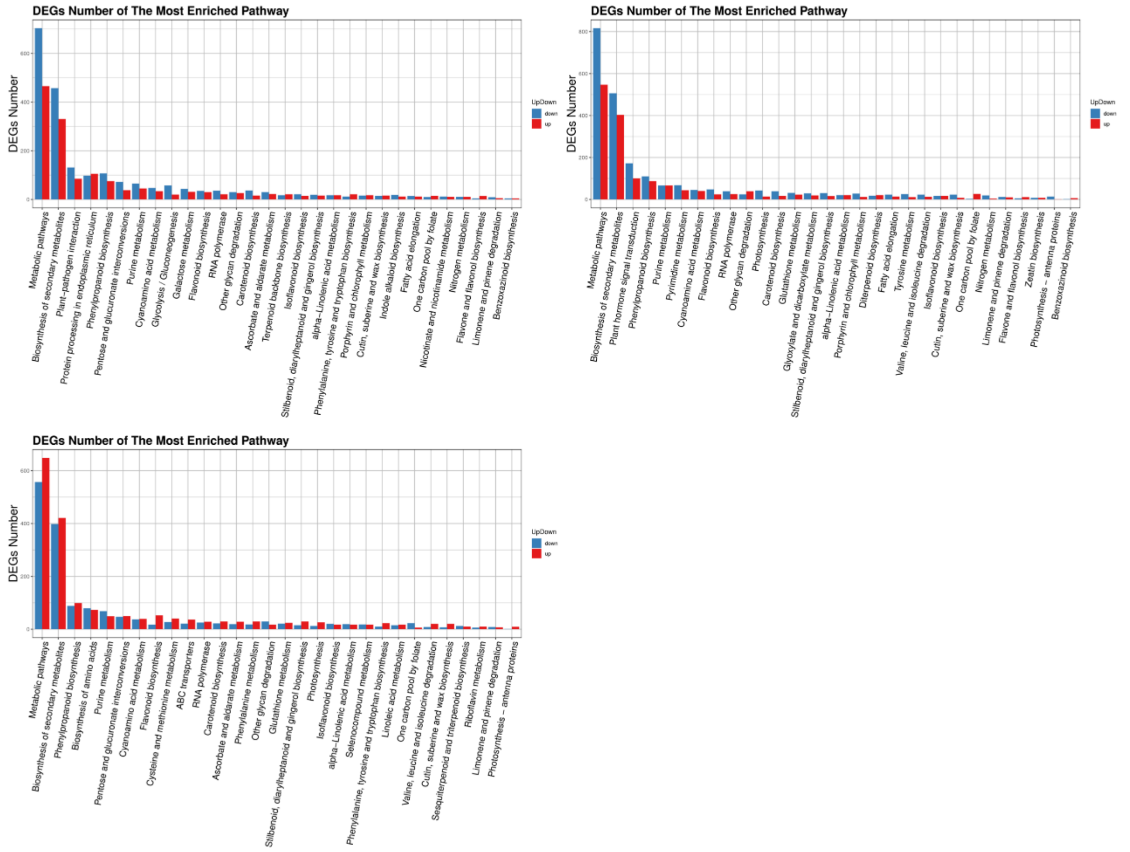

Fig. 19 Enrichment analysis of differentially expressed genes in different color petals

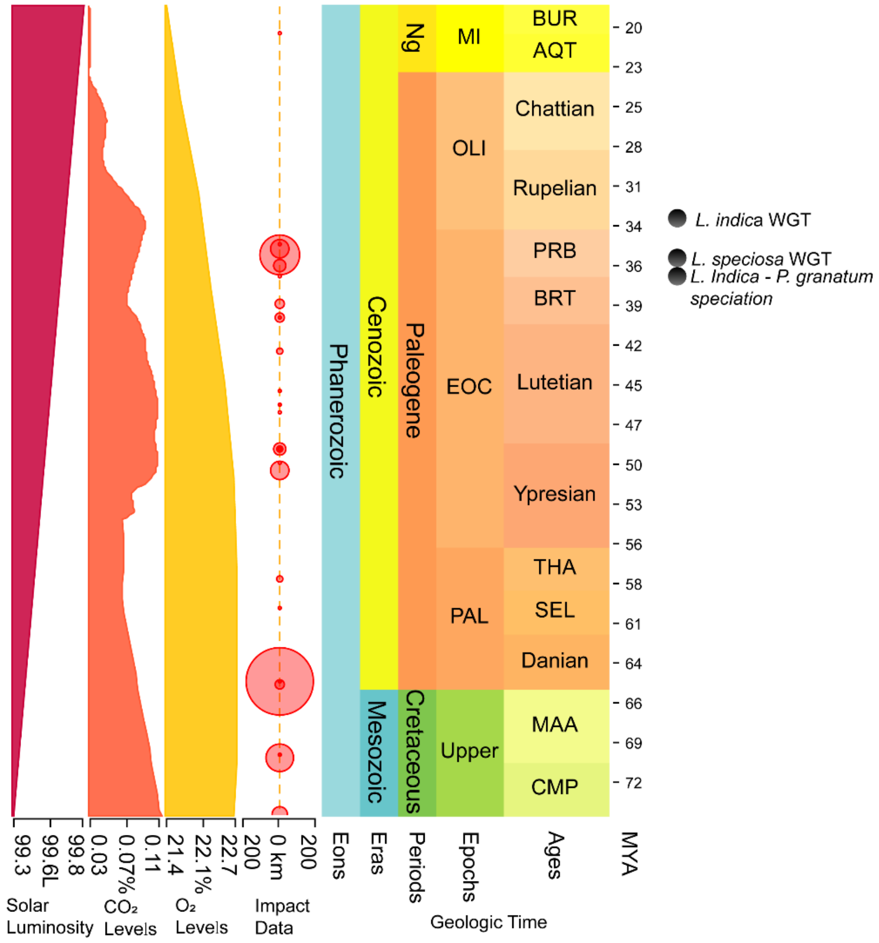

Fig. 20 Climate, geological events and *L. indica* evolution.
